# Microbial community of soda Lake Van as obtained from direct and enriched water, sediment and fish samples

**DOI:** 10.1038/s41598-021-97980-3

**Published:** 2021-09-15

**Authors:** Esra Ersoy Omeroglu, Mert Sudagidan, Mediha Nur Zafer Yurt, Behiye Busra Tasbasi, Elif Esma Acar, Veli Cengiz Ozalp

**Affiliations:** 1 Biology Department, Basic and Industrial Microbiology Section, Faculty of Science, Ege University, 35040 Bornova, Izmir Turkey; 2 KIT-ARGEM R&D Center, Konya Food and Agriculture University, 42080 Meram, Konya Turkey; 3 Department of Medical Biology, Medical School, Atilim University, 06830 Ankara, Turkey

**Keywords:** Ecology, Bacteria, Bacteriology, Microbial communities, Environmental microbiology, Microbial genetics

## Abstract

Soda lakes are saline and alkaline ecosystems that are considered to have existed since the first geological records of the world. These lakes support the growth of ecologically and economically important microorganisms due to their unique geochemistry. Microbiota members of lakes are valuable models to study the link between community structure and abiotic parameters such as pH and salinity. Lake Van is the largest endroheic lake and in this study, bacterial diversity of lake water, sediment, and pearl mullet (inci kefali; *Alburnus tarichi*), an endemic species of fish which are collected from different points of the lake, are studied directly and investigated meticulously using a metabarcoding approach after pre-enrichment. Bacterial community structures were identified using Next Generation Sequencing of the 16S rRNA gene. The analysis revealed that the samples of Lake Van contain high level of bacterial diversity. Direct water samples were dominated by Proteobacteria, Cyanobacteria, and Bacteroidota, on the other hand, pre-enriched water samples were dominated by Proteobacteria and Firmicutes at phylum-level. In direct sediment samples Proteobacteria, whereas in pre-enriched sediment samples Firmicutes and Proteobacteria were determined at highest level. Pre-enriched fish samples were dominated by Proteobacteria and Firmicutes at phylum-level. In this study, microbiota members of Lake Van were identified by taxonomic analysis.

## Introduction

Environments that contain challenging surviving conditions for normal living forms are called extreme environments and microorganisms living in these environments are described as extremophiles. Even though some extremophiles can tolerate the extreme conditions, mostly they need these conditions for their optimal growth. However, a change in conditions causes extremophiles to lose their vitality. Temperature (thermophiles), salinity (halophiles) or pH (alkaliphiles) can be given as examples for distinctive extreme conditions^1^. Since soda lakes are very alkaline lakes with a pH values from 9 to 11 and with salinities (21 g/kg), they are considered as extreme alkaline-saline ecosystems^2,3^. Soda lakes have an unusual geochemical property, they contain many ecological and economically important microorganisms^4^. There are many soda lakes in the world and the most important ones are Lake Bogoria, Lake Nakuru, Lake Elmenteita and Lake Magadi, Kenya; Lakes Abijata, Chitu, Shalla, the East African Rift; Lakes Beseka and Arenguadi, Ethiopia; Lonar Lake, India; Soap Lake, USA; and Lake Manito, Canada^1,4–11^. Among these soda lakes, Lake Van with 1648 m above sea level and exceptional water quality properties, is the biggest lake of Turkey and it is also the largest soda lake in the world. Lake Van is localized in Turkey's East Anatolian high plateau and the lake has maximum depth of 445 m. There are two semi-active volcanoes around Lake Van; the mountain Nemrut, at an altitude of 3050 m above sea level, and the mountain Suphan at an altitude of 3800 m. The last volcanic eruption of Nemrut was reported in 1441. As a result of the chemical erosion of the volcanic rocks and the evaporation process, the lake water is salty (21.4 ‰) and alkaline (155 m mEq^−1^, pH 9.81)^12^. Moreover, Lake Van contains an exceptional endemic fish population. The only type of fish that can adapt to the lake environment is pearl mullet (*A. tarichi*)^13^. Lake Van has the world’s largest microbialites and microbial systems that play an important role in carbonate precipitation^14^. Because of the fact that Lake Van is the largest soda lake, it also serves as a model of the possible high alkaline chemistry of the early ocean^15^. For these reasons, Lake Van attracted the attention of many researchers, however, almost all studies seem to be related to the geological and chemical analysis of the lake^14–20^. Although the geological and chemical features of the lake directly determine the identity of dominant microbiota, the existing microbial population may be also effective in the creation of these features. In this context, the microbial community studies of the lake, as well as the results of the chemical and geochemical studies, are correlated. Studies in extreme habitats like Lake Van have shown that it contains many new genus and species, as well as microorganisms that have properties that can be used in biotechnology industry^4,7^. Especially when considering soda lakes, intense studies with alkaliphilic bacteria attract attention^10,21^. Soda lakes have been the source for many microorganisms important in biotechnological applications^1^. In addition to the newly discovered genus and species^11,22^, new gene regions^23,24^ have been added to the literature as a result of such studies. Among these studies, researchers used culture-dependent and culture-independent methods, or a combination of both^7,8,22^. In this context, culture-dependent methods cannot provide sufficient data for the observation of the whole microbiota due to the high number of non-cultured species. Since such habitats are especially important in investigating evolutionary relationships among living organisms, it is necessary to identify all microbial species. For this reason, to investigate diversity of microbial communities, next generation sequencing (NGS) and high-throughput amplicon sequencing of the 16S rRNA amplicon techniques attracted attention as useful methods^25,26^.

Lake Van has the biggest microbialites and contains an endemic fish species. However, no other study has used 16S rRNA gene metabarcoding to study the microbial diversity of Lake Van. In this study, we aimed to study direct and pre-enriched samples of lake water, sediments, and fish samples collected at different locations in Lake Van in order to identify both the local bacterial community structure and the anthropogenic hygiene indicator bacteria in pre-enriched samples using 16S rRNA NGS.

## Methods

### Site description and sample collection

Lake Van lies in the far east of Turkey in the provinces of Van and Bitlis and is located 1648 m above sea-level in the highlands of eastern Anatolia, Turkey. It is in the location at 38° 37′ N, 42° 50′ E with a depth of 450 m, a surface area of 3522 km^2^ and a volume of 576 km^3^. After Caspian Sea (located between Europa and Asia) and Issyk-Kul (in Kyrgyzstan), it is the third largest (by volume) endorheic or closed body of water on Earth^15^.

The water and sediment samples were taken from the coast of Lake Van, from the lake surface to ten meters depth. For each sample, physicochemical parameters such as pH, temperature, dissolved oxygen concentration, and coordinates of the sampling points were recorded. Water and sediment samples were collected from nine different point of lake in January 2020. Sample codes and their descriptions are given in Table 1. Six fish with different sizes, three from the Gevaş-İnköy area and three from the Edremit region, were obtained from local fish markets in Van city center and transported in sterile sampling bags in cold storage (Table 1).

**Table 1:** Sample types collected from different locations in Lake Van and studied in NGS analysis.

Sample code	Sample type	Direct or pre-enriched DNA extraction applied	Sampling location
DWWS1	Water	Direct DNA extraction	Edremit
DWWS2	Water	Direct DNA extraction	Ergil-1
DWWS5	Water	Direct DNA extraction	Gevaş
DWWS6	Water	Direct DNA extraction	Gevaş-Tatvan
WWS1	Water	Pre-enriched in BHI	Edremit
WWS2	Water	Pre-enriched in BHI	Ergil-1
WWS3	Water	Pre-enriched in BHI	Ergil-2
WWS4	Water	Pre-enriched in BHI	Gevaş (pier)
WWS5	Water	Pre-enriched in BHI	Gevaş
WWS6	Water	Pre-enriched in BHI	Gevaş-Tatvan
WWS7	Water	Pre-enriched in BHI	Gevaş-DSİ-1
WWS8	Water	Pre-enriched in BHI	Gevaş-DSİ-2
WWS9	Water	Pre-enriched in BHI	Akdamar
WSWcontrol	Water	Pre-enriched in Buffered peptone water	Lake Water
WS1K	Sediment	Direct DNA extraction	Edremit
WS2K	Sediment	Direct DNA extraction	Ergil-1
WS3K	Sediment	Direct DNA extraction	Ergil-2
WS5K	Sediment	Direct DNA extraction	Gevaş
WS6K	Sediment	Direct DNA extraction	Gevaş-Tatvan
WS8K	Sediment	Direct DNA extraction	Gevaş-DSİ-2
WS9K	Sediment	Direct DNA extraction	Akdamar
WS1	Sediment	Pre-enriched in Buffered peptone water	Edremit
WS2	Sediment	Pre-enriched in Buffered peptone water	Ergil-1
WS3	Sediment	Pre-enriched in Buffered peptone water	Ergil-2
WS5	Sediment	Pre-enriched in Buffered peptone water	Gevaş
WS6	Sediment	Pre-enriched in Buffered peptone water	Gevaş-Tatvan
WS8	Sediment	Pre-enriched in Buffered peptone water	Gevaş-DSİ-2
WS9	Sediment	Pre-enriched in Buffered peptone water	Akdamar
WS1W	Sediment	Pre-enriched in Buffered peptone water + lake water	Edremit
WS2W	Sediment	Pre-enriched in Buffered peptone water + lake water	Ergil-1
WS3W	Sediment	Pre-enriched in Buffered peptone water + lake water	Ergil-2
WS5W	Sediment	Pre-enriched in Buffered peptone water + lake water	Gevaş
WS6W	Sediment	Pre-enriched in Buffered peptone water + lake water	Gevaş-Tatvan
WS8W	Sediment	Pre-enriched in Buffered peptone water + lake water	Gevaş-DSİ-2
WS9W	Sediment	Pre-enriched in Buffered peptone water + lake water	Akdamar
VB1	Fish-1 mouth	Pre-enriched in BHI	Edremit
VB2	Fish-1 outer surface	Pre-enriched in BHI	Edremit
VB3	Fish-1 intestine	Pre-enriched in BHI	Edremit
VB4	Fish-1 intestinal fluid	Pre-enriched in BHI	Edremit
VB5	Fish-1 visceral organ	Pre-enriched in BHI	Edremit
VB6	Fish-2 mouth	Pre-enriched in BHI	Edremit
VB7	Fish-2 outer surface	Pre-enriched in BHI	Edremit
VB8	Fish-2 intestine	Pre-enriched in BHI	Edremit
VB9	Fish-2 intestinal fluid	Pre-enriched in BHI	Edremit
VB10	Fish-2 visceral organ	Pre-enriched in BHI	Edremit
VB11	Fish-3 mouth	Pre-enriched in BHI	Edremit
VB12	Fish-3 outer surface	Pre-enriched in BHI	Edremit
VB13	Fish-3 intestine	Pre-enriched in BHI	Edremit
VB14	Fish-3 visceral organ	Pre-enriched in BHI	Edremit
VB15	Fish-4 mouth	Pre-enriched in BHI	Gevaş-İnköy
VB16	Fish-4 outer surface	Pre-enriched in BHI	Gevaş-İnköy
VB17	Fish-4 intestine	Pre-enriched in BHI	Gevaş-İnköy
VB18	Fish-4 visceral organ	Pre-enriched in BHI	Gevaş-İnköy
VB19	Fish-5 mouth	Pre-enriched in BHI	Gevaş-İnköy
VB20	Fish-5 outer surface	Pre-enriched in BHI	Gevaş-İnköy
VB21	Fish-5 intestine	Pre-enriched in BHI	Gevaş-İnköy
VB22	Fish-5 visceral organ	Pre-enriched in BHI	Gevaş-İnköy
VB23	Fish-6 mouth	Pre-enriched in BHI	Gevaş-İnköy
VB24	Fish-6 outer surface	Pre-enriched in BHI	Gevaş-İnköy
VB25	Fish-6 intestine	Pre-enriched in BHI	Gevaş-İnköy
VB26	Fish-6 visceral organ	Pre-enriched in BHI	Gevaş-İnköy
VB27	Fish-6 hard roe	Pre-enriched in BHI	Gevaş-İnköy

### DNA extraction and quantification

Direct DNA extraction from water and sediment samples were performed using QIAamp Power Fecal DNA kit (12,830, Qiagen, Germany) as described by the manufacturer. Collected 500 ml water samples (WWS1–WWS9, n:9) were filtered by sterile vacuum filtration system (Sartolab RF500, Sartorius, Germany) containing sterile Whatmann No.1 filter paper to concentrate the lake water and collect microorganisms. The residue on the surface of filter was collected by a sterile swab and inoculated into 10 ml Brain Heart Infusion broth (BHI, Oxoid CM1135B, UK) and incubated at 35 °C for 20 h with shaking at 200 rpm to enrich fastidious bacteria present in lake water. Sediment samples (WS1–WS9, n:7) of 5 g were pre-enriched in 45 ml buffered peptone water (Oxoid CM0509B) and (WS1W–WS9W, n:7) 5 g sediment sample was pre-enriched in 5 ml buffered peptone water supplemented with 40 ml filtered lake water by 0.22 µm filter at 30 °C for 24 h with shaking at 200 rpm. Fish samples (VB1–VB27, n:27) were collected by taking swabs from outer surfaces, inside mouth, intestines, and visceral organs (Table 1). The swabs were used to inoculate 10 ml BHI for pre-enrichment of fastidious bacteria which is present on fish surfaces and inside intestines. The total DNA extraction were carried out from all pre-enriched cultures by phenol/chloroform/isoamyl alcohol method^27^ and the extracted DNA samples were dissolved in sterile DNase/RNase free ultrapure water. Quantity and purity of all extracted total DNA samples were determined using Take3 plate spectrophotometrically (EPOCH-2, BioTek, USA) and DNA samples were stored at − 20 °C.

### NGS and metabarcoding

NGS method was used to identify bacterial communities. 16S metabarcoding library preparation was carried out according to the manufacturer’s instructions as described in document Part # 15044223 Rev. B (Illumina, Inc., California, USA). An amplicon PCR was performed by 12.5 ng total DNA previously diluted to 5 ng/µl concentration with sterile ultrapure water. The forward and reverse primers (1 µM) included overhang adapter sequences (Forward primer.

5′-TCGTCGGCAGCGTCAGATGTGTATAAGAGACAGCCTACGGGNGGCWGCAG-3′ and reverse primer 5′-GTCTCGTGGGCTCGGAGATGTGTATAAGAGACAGGACTACHVGGGTATCTAATCC-3′) were used to amplify V3 and V4 regions of prokaryotic 16S rRNA regions by 2 × KAPA HiFi HotStart Ready Mix (KK2602, 07958935001, Roche, Germany) in total 25 µl amplicon PCR mixture. The amplicons were analyzed in 1.5% (w/v) agarose gel electrophoresis. Addition of dual index sequences (N701-N715 and S502-S511) to amplified regions in amplicon PCR by Index PCR were performed using the Nextera XT index Kit v2 Set-A (15052163, Illumina). Amplicon PCR and index PCR products were cleaned using AMPure XP beads (A63881, Beckman Coulter, USA) on magnetic racks. Cleaned DNA samples were quantified using AccuBlue NextGen dsDNA Quantitation kit (31060, Biotium, Inc. USA) as described by manufacturer’s instructions using multimode plate reader (Mithras^2^ LB943, Berthold, Germany). All samples were diluted to 10 nM with sterile ultrapure water and equimolar portions of samples were pooled in one tube. The obtained DNA library was finally diluted to 35 pM and 5% (v/v) PhiX control v3 (15017666, Illumina) was added to library that was used as a control DNA. After that, 20 µl library containing PhiX DNA was loaded to iSeq 100 i1 Cartridge (300 cycle). The sequencing was performed in iSeq 100 system (Illumina, Inc.) by pair end read type and two reads of 151 bp read length. The obtained forward sequences were analyzed by operational taxonomic unit (OTU) approach using SILVA NGS 1.4 software with reference version 138.1 and SINA v1.2.10 for ARB SVN (revision 21008) with BLASTn 2.2.30 + at phylum and family-level^28–34^. Shannon diversity index was also determined by SILVA NGS software^28^. Principal components analysis (PCA) with varimax rotation and construction of a dendrogram using single linkage were performed using IBM SPSS Statistics program version 22.0 (2020). Canonical Correspondence Analysis (CCA) was applied using the PAleontological STatistics (PAST) Software version 4.06b package (2021) to consider environmental variables temperature, pH and dissolved oxygen levels in lake water and sediment samples^35^.

## Results

### Physicochemical parameters

During sampling, variations were observed about temperature of sampling locations even though all samples were collected in the same day. In fact, Lake Van covers a large area and temperature values can depend on weather conditions. The pH values of the lake water ranged from 9.38 to 9.57. Only one pH value of 7.9 were recorded at a sampling location where lake soda water and fresh water were mixed. In all sampling locations, similar dissolved O_2_ values were recorded (Table 2).

**Table 2:** Physicochemical parameters of lake water and sediment samples.

Sampling location	Sample code*	Sampling date	Depth (m)	Coordinate**	Temperature (°C) ***	pH***	Dissolved O_2_ (mg/l) ***
Edremit	WWS-1 water	01/18/2020	7–10	38° 25′ 24,90″ N 43° 16′ 15,84″ E	6.1	9.55	4.0
	WS-1 Sediment						
Ergil-1	WWS-2 water		0–1	38° 23′ 28,74″ N 43° 11′ 17,23″ E	9.2	9.57	4.2
	WS-2 Sediment						
Ergil-2	WWS-3 water		0–1	38° 23′ 28,74″ N 43° 11′ 17,23″ E	8.1	7.90	4.2
	WS-3 Sediment						
Gevaş (pier)	WWS-4 water		3	38° 18′ 52,25″ N 43° 06′ 58,74″ E	7.8	9.56	4.1
Gevaş	WWS-5 water		7–10	38° 18′ 53,67″ N 43° 07′ 27,46″ E	7.8	9.38	4.1
	WS-5 Sediment						
Gevaş-Tatvan	WWS-6 water			38° 19′ 07,46″ N 43° 05′ 33,18″ E	5.5	9.56	4.1
	WS-6 Sediment						
Gevaş-DSİ-1	WWS-7 water			38° 18′ 49,97″ N 43° 04′ 34,95″ E	5.5	9.56	4.0
Gevaş-DSİ-2	WWS-8 water		0–1	38° 18′ 41,18″ N 43° 03′ 56,69″ E	4.7	9.56	4.0
	WS-8 Sediment						
Akdamar	WWS-9 water			38° 18′ 45,00″ N 43° 03′ 24,22″ E	6.4	9.56	3.9
	WS-9 Sediment						

*WWS: Water sample, WS: Sediment sample collected from Lake Van.

**iGPS was used.

***HQ40d multi LDO101 and HQ40d multi PHC101 were used.

### NGS analysis

**Figure 1:**
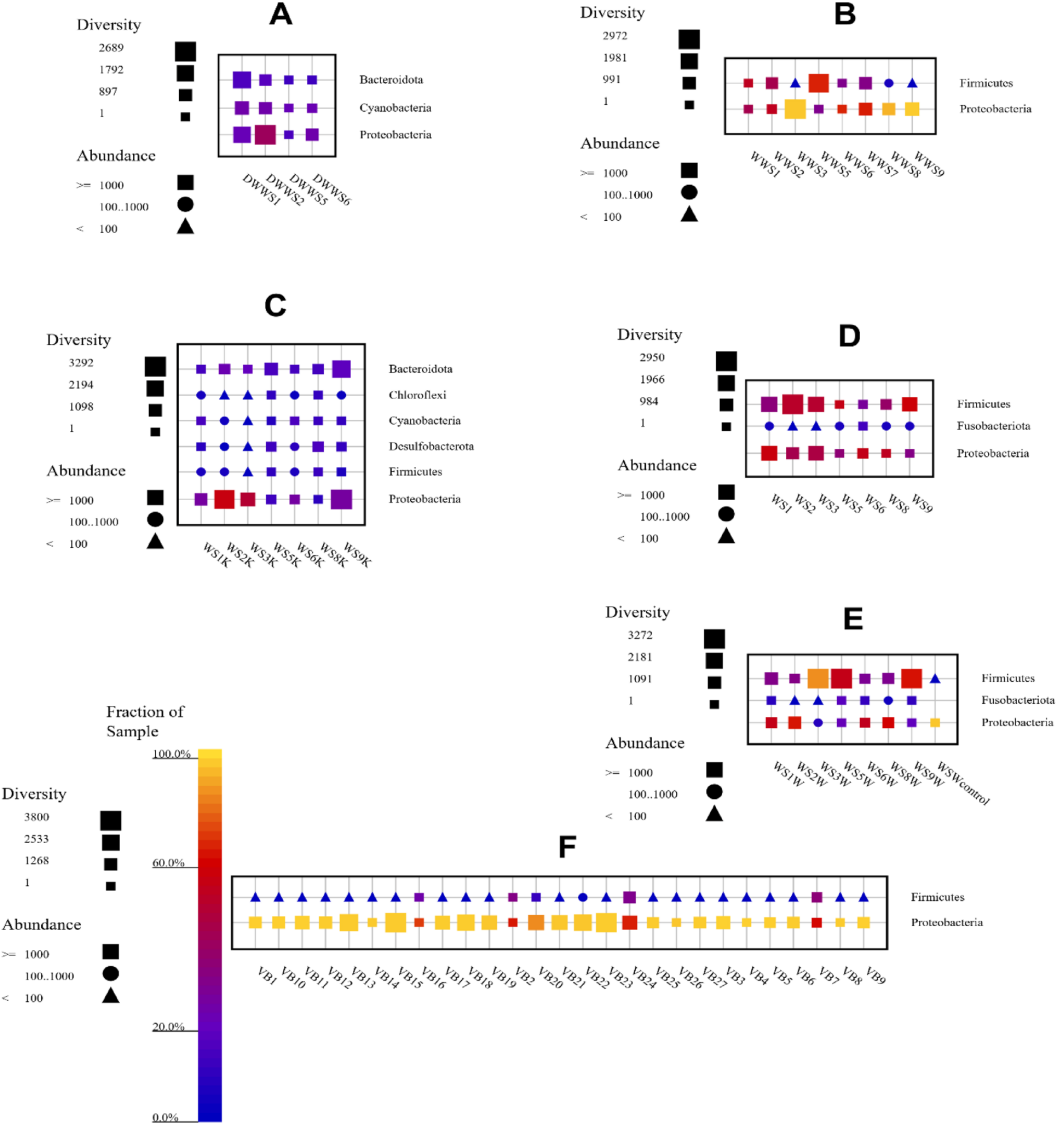
Taxonomic fingerprint of (**A**) directly studied water samples (DWWS1–DWWS6), (**B**) pre-enriched water samples (WWS1–WWS9), (**C**) directly studied sediment samples (WS1K–WS9K), (**D**) pre-enriched sediment samples in buffered peptone water (WS1–WS9), (**E**) pre-enriched sediment samples in buffered peptone water supplemented with lake water (WS1W–WS9W), (**F**) pre-enriched fish samples (VB1–VB27) of Lake Van at phylum-level (The figure was created using SILVA NGS 1.4 v138.1).

**Figure 2:**
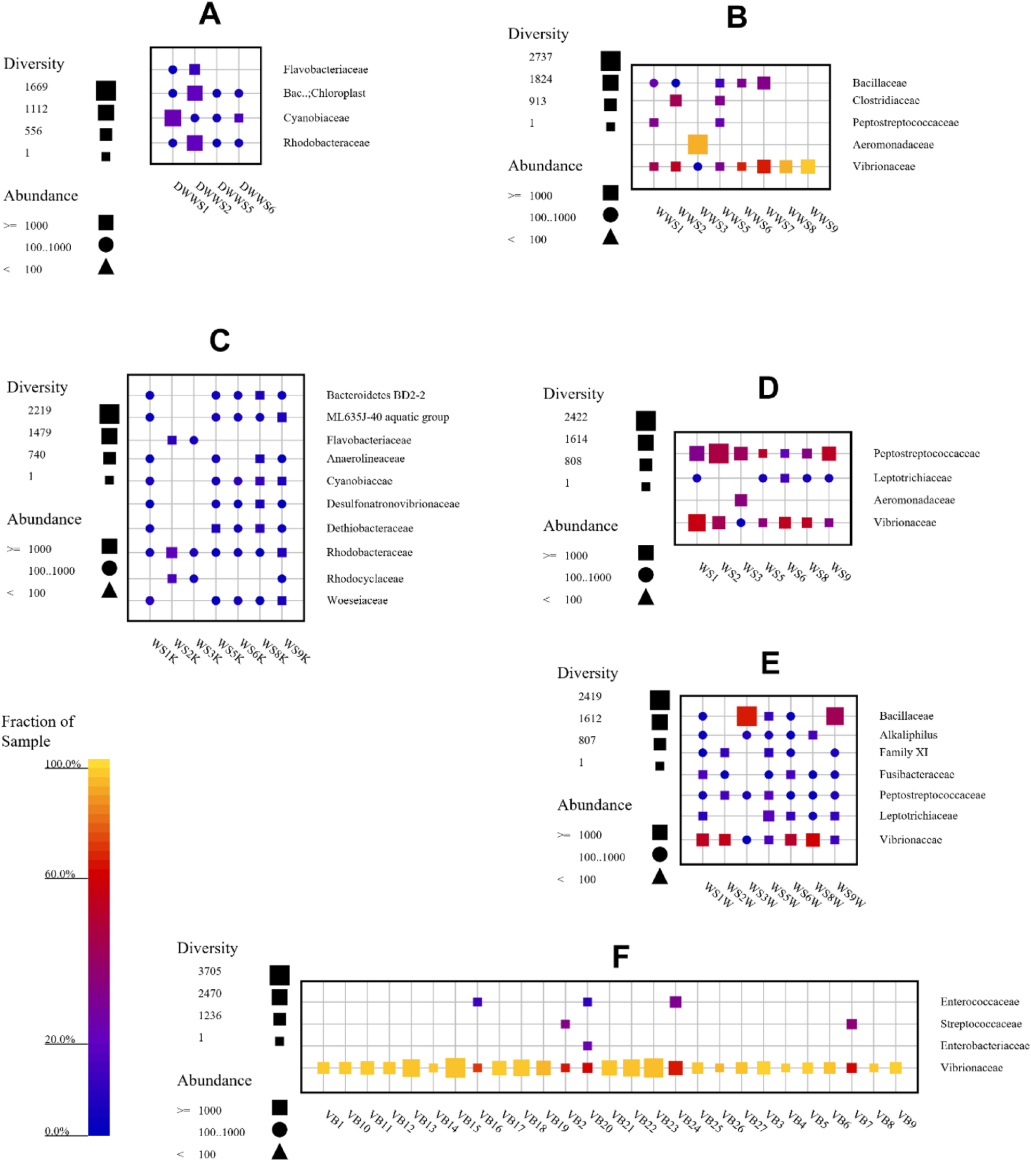
Family-level taxonomic fingerprint of (**A**) directly studied water samples (DWWS1–DWWS6), (**B**) pre-enriched water samples (WWS1–WWS9), (**C**) directly studied sediment samples (WS1K–WS9K), (**D**) pre-enriched sediment samples in buffered peptone water (WS1–WS9), (**E**) pre-enriched sediment samples in buffered peptone water supplemented with lake water (WS1W–WS9W), (**F**) pre-enriched fish samples (VB1–VB27) of Lake Van (The figure was created using SILVA NGS 1.4 v138.1).

Metabarcoding analysis of water, sediment and fish samples demonstrated that Bacteria domain was dominant in all directly studied and pre-enriched samples. The data obtained from 16S rRNA NGS were analyzed with OTU approach for taxonomic classification (see supplementary information Figs. S1, S6 at phylum-level and Figs. S7, S12 at family-level taxonomic analysis results). Direct DNA extraction from eight water samples resulted in amplification of desired 16S rRNA region only in four water samples (DWWS1, DWWS2, DWWS5, and DWWS6). However, in pre-enrichment applied water samples (WWS1–WWS9), eight of nine samples were amplified and sequenced. Only DNA extracted from pre-enriched WWS4 collected from Gevaş (pier) could not be amplified. Bacterial communities, especially fastidious bacteria, present in water were enriched by collecting residues on the surface of filter. NGS results showed that direct DNA extracted water samples (n:4) revealed 82,247 reads (47,287 classified sequences, 14,971 clustered sequences, and 32,798 OTUs) and 136,754 reads (85,118 classified sequences, 24,967 clustered sequences, and 23,151 OTUs) were recorded in pre-enriched water samples (n:8). In bacterial community structure of directly studied water samples, Proteobacteria, Bacteroidota, and Cyanobacteria were identified at phylum-level (Fig. 1A). In the case of pre-enriched water samples, Proteobacteria and Firmicutes were identified (Fig. 1B). Cyanobiaceae and Rhodobacteraceae were determined at family-level of direct water samples (Fig. 2A). Whereas, pre-enriched water samples were dominated by Vibrionaceae and Aeromonadaceae at family-level taxonomic classification. Among all the other locations, Gevaş location (WWS5) showed the highest Firmicutes population. In fact, pre-enrichment especially increased number of Firmicutes and fastidious bacteria present in lake water or contaminated by human or animal sources due to agricultural facilities around lake or by the reaching rivers to Lake Van.

Direct DNA extraction from seven sediment samples using a commercially available kit results showed that 155,797 reads (91,649 classified sequences, 28,326 clustered sequences, and 66,798 OTUs) were obtained in NGS and all samples were dominated by Proteobacteria and Bacteriodota. In addition, Cyanobacteria, Desulfobacterota, Firmicutes, and Chloroflexi were also identified at phylum-level (Fig. 1C). In pre-enriched sediment samples with peptone water (WS1–WS9) 154,470 reads (90,296 classified sequences, 28,488 clustered sequences, and 28,999 OTUs) and peptone water supplemented with sterile lake water (WS1W–WS9W), 158,725 reads (86,526 classified sequences, 28,605 clustered sequences, and 33,764 OTUs) were recorded in NGS analysis. Taxonomic classification showed that all pre-enriched sediment samples were dominated by Firmicutes and Proteobacteria (Fig. 1D). Highest populations were observed in WS3W, WS5W, WS2, and WS9W samples (Fig. 1D,E). In addition, Fusobacteriota were also abundant in sediment samples. Directly studied sediment samples were dominated by Rhodobacteraceae, Dethiobacteraceae, and Cyanobiaceae, (Fig. 2C) whereas, pre-enriched sediment samples were dominated by Vibrionaceae, Peptostreptococcaceae, and Bacillaceae at family-level taxonomic classification (Fig. 2D,E). Interestingly, in WSWcontrol sample, the number of Proteobacteria was highest with respect to all other pre-enriched sediment samples and this indicated that like directly studied samples water contain its own microbiota in addition to pre-enrichment. In fact, the result of WSWcontrol sample showed the free-living community in lake water pass through filtration.

**Figure 3:**
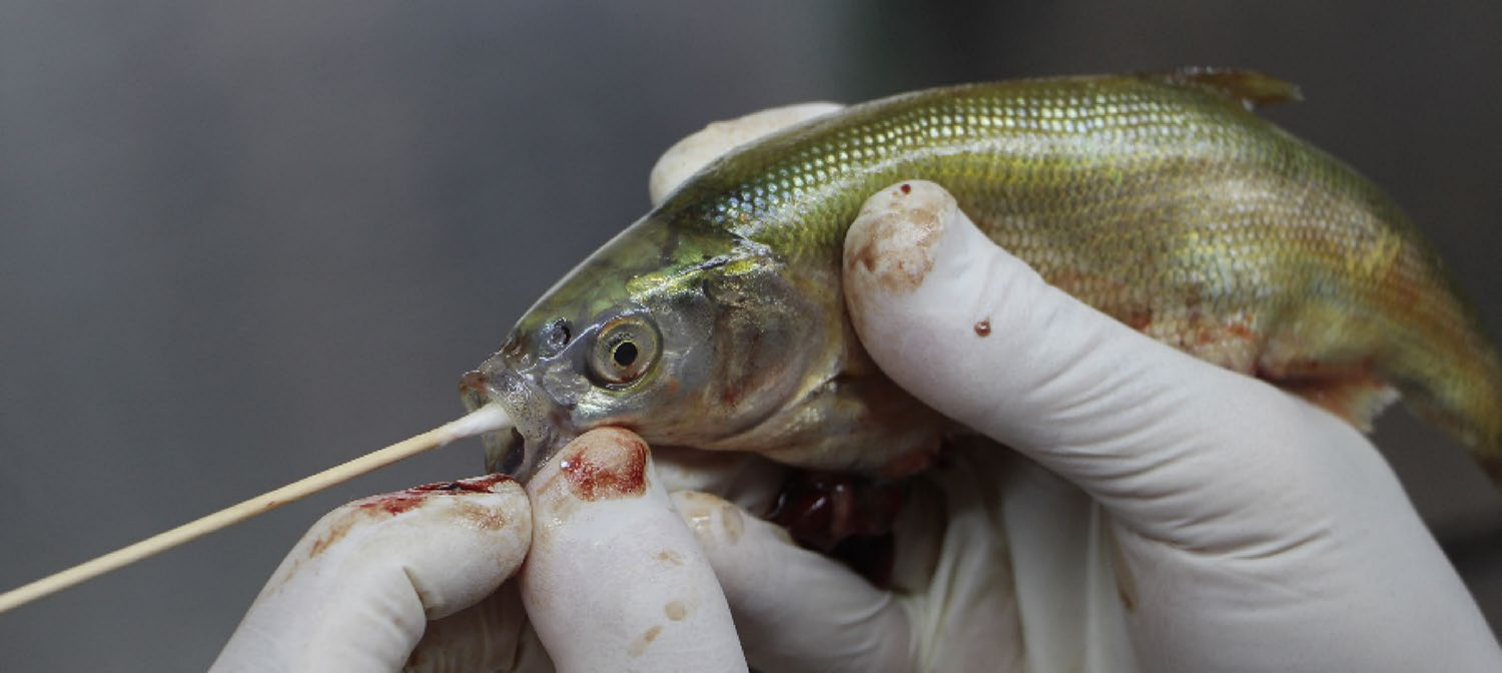
Sampling by a swab from a pearl mullet mouth.

*A. tarichi* adopt to live in alkaline pH environment and saline lake water (Fig. 3). The fish samples taken from interior part of mouth, outer surface, and inner part of intestines, intestinal fluid, and surface of visceral organs were pre-enriched in BHI to increase number of bacteria to extract sufficient amount of DNA for amplicon PCR experiments. In NGS analysis, 576,884 reads in total (354,563 classified sequences, 86,459 clustered sequences, and 78,610 OTUs) were obtained in 27 samples from collected six pearl mullet fish (Table 1). All pre-enriched samples were dominated by Proteobacteria and Firmicutes at phylum-level. Moreover, in VB24, VB7, VB2, VB16, and VB20 samples, all of them obtained from outer fish surface, Firmicutes were abundant with respect to other fish samples (Fig. 1F). At family-level taxonomic classification, all fish samples were dominated by Vibrionaceae (Fig. 2F). In our opinion, this indicates that microbiota members of lake water which belong to Firmicutes may adhere or form a biofilm on surface of pearl mullet fish outer surface and this may help the adaptation or survival of fish in this extreme environment.

### Diversity of samples

Alpha diversity measuring richness of bacterial communities of lake was determined by Shannon diversity values. Directly studied water and sediment samples showed higher diversity values for Shannon diversity index than pre-enriched samples of both water and sediment as well as pre-enriched fish samples (see supplementary information Table S1). The highest alpha diversity value (13.75) was recorded in WS5K directly studied sediment sample obtained from Gevaş and the lowest values was 3.20 in VB23 pre-enriched fish sample from mouth of fish obtained from Gevaş-İnköy. PCA analysis of directly studied water and sediment samples from sequencing data showed that a separation was observed between two communities (see supplementary information Fig. S13A, S14, S15 and S16). The dendrogram using average linkage (within groups) of water and sediment samples of Lake Van indicated that water samples were closely related, but sediments samples were separated to two groups and WS5K collected from Gevaş and WS8K collected from Gevaş-DSİ-2 were distinct from other sediment samples (see supplementary information Fig. S13B). Furthermore, on the basis of PCA pre-enriched water, sediment and fish samples indicated that all samples were closely related (see supplementary information Fig. S17A). Two dimensional PCA results of pre-enriched samples (see supplementary information Fig. S18, S20) showed that there was no separation of water, sediment or fish samples from each other groups. The dendrogram of pre-enriched samples indicated that fish samples were closely related with both water and sediment samples (see supplementary information Fig. S17B). Upon this finding, it was considered that the main source of lake microbiota was sediment. All geochemical parameters (ions, salts, elements, etc.) and organic matters settled at the bottom of lake could provide essential nutrients for sufficient growth of microorganisms in aerobic and anaerobic environments of lake.

**Figure 4:**
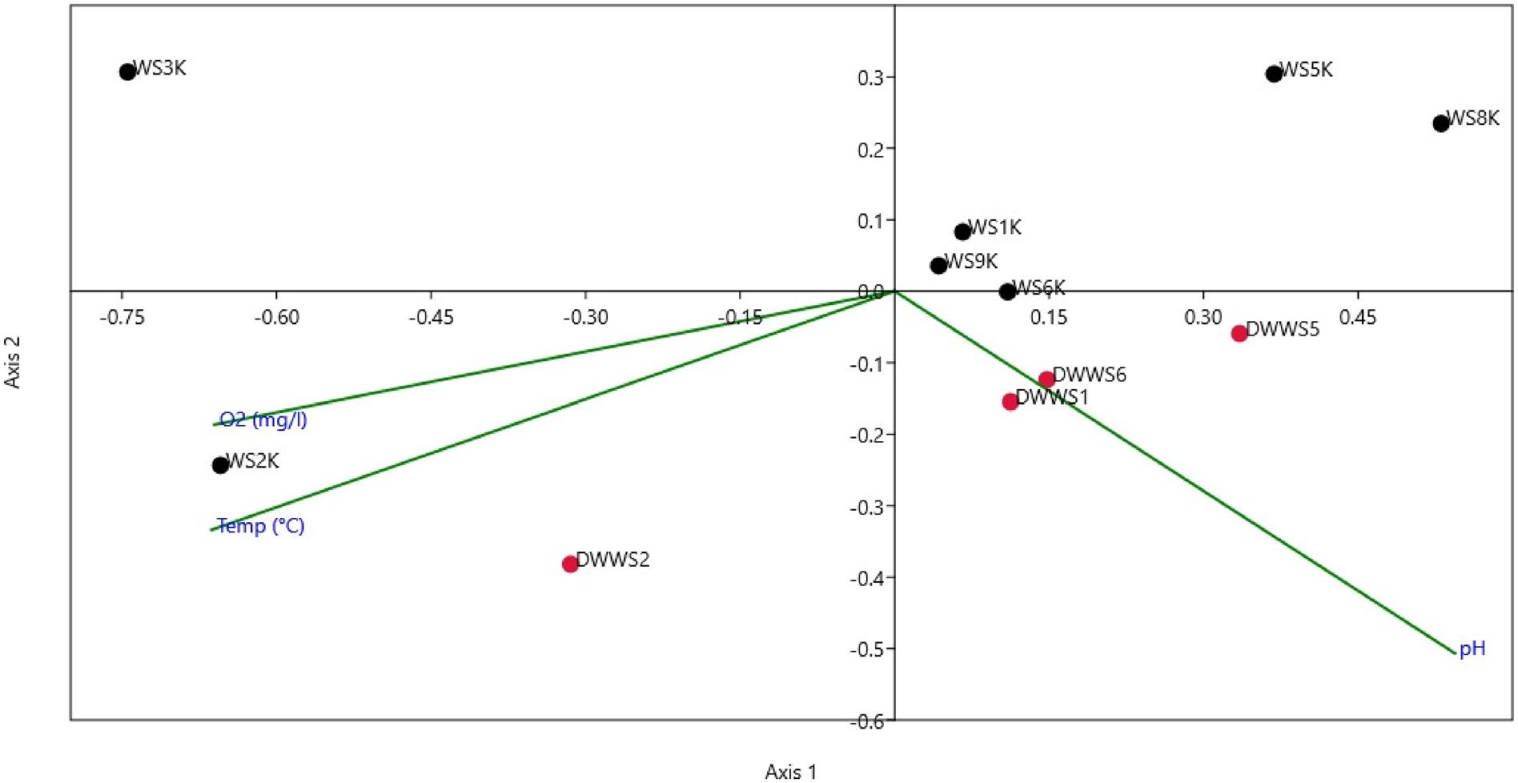
CCA result of directly studied water (DWWS1–DWWS6, red circles) and sediment (WS1K–WS9K, black circles) with environmental variables temperature, pH and dissolved oxygen concentrations at sampling locations in Lake Van (The figure was created using the PAST software package version 4.06b).

CCA analysis was applied to determine the relationships between collected water and sediment samples and environmental variables like water temperature, pH and dissolved oxygen concentrations in Lake Van. Microbial diversity in directly studied water samples (DWWS1, DWWS5 and DWWS6) was related to pH, whereas water sample (DWWS2) and sediment sample (WS2K) both collected from Ergil-1 location (Table 2) correlates with pH values and dissolved oxygen concentrations (Fig. 4). Other sediment samples did not associated with tested environmental variables.

## Discussion

Soda lakes are classified as extreme environments in terms of both alkalinity and salinity, thus the microbiota living in these habitats and their physiological roles in various ecological cycles have always been at the center of many researcher’s attention. Considering the special conditions of these habitats, culture-independent techniques, especially high throughput sequencing, have become increasingly important due to the inadequacy of microbiota determination via traditional culture-based techniques. For this reason, recent studies on culture-independent microbial community have intensified on soda lakes, which are the habitat of new species as well as new gene sources^24,36–39^. Although extensive studies have been conducted on various soda lakes^1,7–9,11,25,40^, it is observed that no comprehensive study has been carried out regarding Lake Van. In this study, DNA samples, extracted directly from water and sediments as well as from short-term pre-enriched water, sediment, and fish samples, were used to elucidate microbial diversity of Lake Van by a metabarcoding approach.

### Physicochemical properties of lake water

The salinity of lake is 21.4 g/kg and the pH value is around 9.7–9.8^14^. Our observations showed that pH was 9.38–9.57 in lake water, however only in Ergil-2 location, pH was recorded as 7.90. In this location, freshwater supplementation to lake was observed. The samples were collected in winter (Jan. 2020) and the water temperature of lake varied from 4.7 to 9.2 °C. The water was warmer near surface than in depth. Dissolved oxygen concentration values of water samples were almost the same (3.9–4.2 mg/l) in all locations. Yigit et al. (2017)^41^ examined water samples collected from eight different points and nine different depths in Lake Van and average pH values were recorded as 9.52. Main cation was sodium with average 8612.6 ppm and main anions were chloride 8703.5 ppm and sulphate average 2900.4 ppm concentrations. Another study was performed to analyze physicochemical properties of Lake Van water showed that average pH was 9.54 and main cations were sodium 7673.15 ppm and potassium 524.60 ppm, moreover main anions were chlorine 5320.13 ppm and sulphate 2466.36 ppm concentrations^42^. In a recent study, heavy metal (Al, B, Cd, Co, Cr, Cu, Fe, Mn, Ni, Pb, and Zn) concentrations were measured in surface water of Lake Van and the results indicated that Cr and Cu were dominant in lake water especially in coastal part of Edremit wastewater treatment plant^43^. At the same time, in another study; as a result of seasonal water and sediment sampling from Lake Van, it was determined that the concentrations of arsenic classified as human carcinogen varied, reaching the highest concentrations in autumn, with 26.070 µg/kg in sediment and 261 µg/kg in lake water^44,45^.

### Bacterial diversity of water, sediment, and fish samples

Geological and hydrochemical properties of Lake Van and all rivers feeding the lake have been previously investigated^15,16,18–20,46^. Additionally, characterization of microbialite were carried out by various researchers^14,47–49^. In this study, the data from community analysis shows that Bacteria domain were dominant in water, sediment, and fish samples of Lake Van rather than the Archaea domain. When we looked at all the water samples, we realized that Proteobacteria were dominant at phylum-level. In the study conducted by Lopez-Garcia et al. (2005)^49^, it showed that Bacteria domain was dominant in the microbialite microbiota of Lake Van, Firmicutes was located as the dominant phylum, and other common bacteria were the members of Proteobacteria, Cyanobacteria, and Actinobacteria. It was also determined that there was a large amount of Cyanobacteria members in microbialite samples taken from different locations and from a depth of approximately 8–10 m of Lake Van^14^. Microbial populations present in water of Lake Van have been previously examined and a NGS study was performed with a water sample taken from only one point. As a result of this study, it was determined that Proteobacteria, Actinobacteria, and Verrucomicrobia were dominant at phylum-level^50^. However, there is a known fact that sampling is essential in microbiology and sampling must be done to represent the entire habitat to determine the actual microbial load and diversity. For this reason, our study consisting of sampling was performed at nine different locations (Table 2) to represent whole lake microbiota. Because of a fact, Lake Van is fed by rivers such as Engil Stream, Küçüksu and Karasu as well as containing regions with anthropogenic density^15^. This situation causes alterations on hydrochemical properties of the lake, especially in pH and it also influences microbial diversity. As a matter of fact, when the water samples were taken from Van, Tatvan, Ahlat, and Edremit, which are located in the borders of Lake Van, it was demonstrated that there were some alterations in ionic properties of water, especially in pH and salinity values^51^. Moreover, the diversity of alkaliphilic bacteria based on culture-dependent and culture-independent techniques such as DGGE (Denaturing Gradient Gel Electrophoresis) and FISH (Fluorescence In-Situ Hybridization) were carried out and *Halomonas*, *Alkalimonas*, *Marinobacter*, *Vibrio*, *Rhodococcus*, *Pseudomonas,* and *Alteromonadales* were found to be dominant members of the lake microbiota^51^. In our study, besides Cyanobiaceae, Rhodobacteraceae, Cyanobacteria (Chloroplast), and Flavobacteriaceae were dominant in directly studied water samples, pre-enriched water samples were dominated by Vibrionaceae, Aeromonadaceae, Bacillaceae, Clostridiaceae, and Peptostreptococcaceae at family-level (Fig. 2). In directly studied sediment samples, Rhodobacteraceae were dominant, whereas in pre-enriched sediment samples Vibrionaceae, Peptostreptococcaceae, Bacillaceae, Leptotrichiaceae, Fusibacteraceae, and Alkaliphilus were identified with high abundance (Fig. 2 and see supplementary information Fig. S10, S11).

Pearl mullet is an endemic fish species survive in alkaline lake water and that is also economically important for local fisheries^52^. Danulat and Kempe (1992)^53^ carried out a study about the physiology of pearl mullet and Bostancı and Polat (2011)^54^ analysed 240 *A. tarichi* specimens collected from Lake Van to investigate their age, length, and weight. In our study, we analyzed six pearl mullet samples collected from local fish markets in Van on the basis of their microbiota present on surface and in organs especially intestines. Vibrionaceae was dominant in all pre-enriched fish samples at family-level (Fig. 2). Interestingly, abundance of Enterococcaceae, Streptococcaceae, and Enterobacteriaceae were also determined on outer surface of fish samples (VB2, VB7, VB16, VB20, and VB24) (see supplementary information Fig. S12). This was linked to anthropogenic contamination of lake water and these bacteria can adhere and form biofilm on fish surface. Furthermore, the habitat of the Armenian gull (*Larus armenicus*) is Lake Van, small islands in lake and its coastal surroundings^55^. This gull can carry main contaminant microorganisms to the lake with their feces. Bilgili et al. (1995)^42^ analyzed residues of heavy metals in 160 fish samples collected from Lake Van and their results indicated that high level of zinc, iron, and copper were found. Microbiota members of fish in the lake could help to survive with heavy metal resistance. At this point, more research is needed to elucidate heavy metal resistance in fish^13,42,52^.

Classical microbiology techniques have been used intensively for a long time, especially for the discovery of unique extremophilic microbial communities that can survive in soda lakes^22^. Lots of microbiota members living in such habitats have still not been unearthed due to the insufficiency of our current cultivation knowledge. This makes it necessary to use more effective diagnostic techniques to discover new microorganisms and to understand their physiology in order to elucidate their roles in the ecosystem. In this context, metabarcoding studies are carried out to perform high throughput sequencing process directly from environmental samples. The studies have been carried out in soda lakes such as Big Soda Lake^56^, Mono Lake^57^, Searles Lake^58^, Lake Bogoria^59^, Lake Nakuru^60^, Kulunda Steppe^61^ and Lake Um-Risha^62^ to identify microbial diversity. Considering the studies examining the microbiota of soda lakes, as well as our study, members of Bacteria appear to be dominant in these studies. Members of Proteobacteria, Firmicutes, Bacteriodetes, Actinobacteria, Spirochaetae, and Cyanobacteria were common bacteria at phylum-level to constitute the microbiota of the soda lakes^22^. In fact, the majority of nitrogen fixators in soda lakes are members of Cyanobacteria^10^ and as a result of our study, the members of Cyanobacteria were found in directly studied water and sediment samples. Cyanobacteria have traditionally been considered the only diazotrophic component of the oxygenic phototrophic community. However, these species are salt-tolerant and the mechanism of primary nitrogen fixation in hypersaline soda lake environment remains as a mystery^22^. Cyanobacteria and anoxygenic phototrophic Bacteria dominated African soda lakes. Additionally, Gammaproteobacteria, Firmicutes and Actinobacteria members were isolated and characterized by biochemical and molecular methods^6^. Mwrichia et al. (2011)^63^ studied an African soda lake Elmenteita and identified 37 orders in Bacteria domain. Although Firmicutes especially *Bacillus*, *Lactobacillus*, and *Clostridium* genera were found in sediment samples, water samples of the lake were dominated by Proteobacteria with methylotrophs and non-sulphur phototrophs (Alphaproteobacteria), hydrogenotrophic Bacteria and methylotrophs (Betaproteobacteria), nitrifiers, sulfur oxidisers and anoxygenic phototrophs (Gammaproteobacteria). They also indicated that Bacteroidetes and Spirochaetes were common in all East African soda lake sediments. Vavourakis et al. (2018)^40^ reported four surface sediments of Siberian soda lakes with pH 10 and 70–400 g/l salt content. In that study, three bacterial groups (Firmicutes, Bacteriodetes, and Gammaproteobacteria) were dominant in varying ratios in the examined samples. Additionally, bacterioplanktons were also identified that live in soda lakes containing different proportions of salt. Although the dominant microbiota members belong to the same phyla, the salt concentration in the sedimentary pore water of the soda lakes affect the microbial community composition^64^.

This study reveals that pre-enrichment has a reducing effect on species diversity as seen from Shannon diversity index values (see supplementary information Table S1). This might be due to the media used (brain heart infusion broth and buffered peptone water) in the pre-enrichment. They can favor fastidious bacteria originated from human or animal sources (e.g. armenian gulls) transported by rivers or contaminated from coastal parts of the lake. In fact, some bacterial species may not be metabolically suitable for the nutrients in the pre-enrichment medium or whose cultivation conditions cannot be provided in these environments^65^. For this reason, NGS studies performing direct DNA isolation applied samples are important in terms of showing the real microbial diversity.

In our study, the enrichment treatments select for Firmicutes over Bacteroidota in water and sediment samples. Similarly, sediment and surface water samples collected from Lonar soda lake of Maharashtra state, India were enriched in nutrient broth at pH 10.5, nutrient broth at pH 10.5 with 30 g/l sodium chloride and Tindal’s medium. The grown bacteria were isolated and they were belong to mainly Firmicutes at phylum-level^66^. In directly studied brine and sediment samples from soda lakes or alkaline environments were also found to contain higher numbers of members of Firmicutes than Bacteroidetes. Zhao et al*.*^67^ studied microbiome of soda-saline lakes in Inner Mongolia and they obtained 385 metagenome-assembled genomes (MAGs) and among them 38 MAGs contained the abundant species. Most of the MAGs were shown to belong to the phyla Proteobacteria (119 MAGs), Firmicutes (33 MAGs), Bacteroidetes (29 MAGs) and Actinobacteria (20 MAGs). Moreover, Lavrentyeva et al*.*^68^ studied environmental samples from the Gudzhirganskoe saline lake (Barguzin Valley, Russia) and all sediment samples were dominated by Firmicutes. In another study, Glaring et al*.*^69^ reported microbial diversity of the submarine ikaite columns with less than 6 °C and above pH 10 located in the Ikka Fjord in Southern Greenland. Their sequencing results of the ikaite columns showed that most abundant bacterial phyla were Proteobacteria, Firmicutes, Bacteroidetes, Actinobacteria, and Cyanobacteria.

For the degradation of polymers produced by primary manufacturers, aerobic and anaerobic hydrolytics come into play in the food chain^10^. *Bacillus* and *Clostridium* strains, which are haloalkaliphilic, were determined in water and sediment samples of Lake Van. Apart from this, primary and secondary anaerobic groups such as fermentative, acetogenic, methanogenic and sulfate-reducing bacteria, which can use monomers and oligomers, are well adapted to haloalkaliphilic conditions, are also included in the lake microbiota. As expected, the largest variety in this scope was determined in sediment samples taken from Lake Van. It was determined that fermentative such as *Spirochaeta* acetogenic, *Clostridium*, and sulfate reduction species such as *Desulfosarcina*, *Desulfonatronovibrio* and *Desulfonatronum* were the members of the lake microbiota. It was also found that *Methyloprofundus* (obligat metanotroph) which was isolated and identified from the deep-sea ocean sediment by Tavormina et al*.* for the first time in 2015^70^ was harboured in the intestine contents of pearl mullet and this genus has been characterized by a single species (*M. sedimenti*). Similar situation showed up to be valid for *Desulfurivibrio* (*D. alkaliphilus*)^71^ and *Geopsychrobacter* (*G. electrodiphilus*)^72^, the members of Deltaproteobacteria; *Proteocatella* (*P. sphenisci*)^73^, *Acidaminobacter* (*A. hydrogenoformans*)^74^, *Anaerofustis* (*A. stercorihominis*)^75^, *Anaerovorax* (*A. odorimutans*)^76^ and *Pilibacter* (*P. termitis*)^77^, the members of Firmicutes; *Roseibaca* (*R. ekhonensis*)^78^, the member of Alphaproteobacteria; *Nitriliruptor* (*N. alkaliphilus*)^79^, the member of Actinobacteria, and *Salinispirillum* (*S. marinum*)^80^ and *Buchnera* (*B. aphidicola*)^81^, the members of Gammaproteobacteria which have been characterized by only one species so far. The different microbiota profile that emerged because of the different pre-enrichment processes applied in sediment samples revealed how effective the environmental factors were on the physiology of microorganisms. Although there are some differences in microbial diversity profiles in different soda lakes existing on earth, the similarities existing in terms of photosynthetic primary producers are the most outstanding feature^4^. Current differences are manifested in microbiota members and their numbers as a consequence of local environmental and geochemical impacts.

One of the reasons why researchers show great interest in soda lakes is because that these lakes are in uncharted treasure position for biotechnologists. Because these habitats contain different types of extremophilic microorganisms which have the potential to produce enzymes called extremozymes active at both alkaline pH and high salinity. In industry, alkali-stable extracellular protease, lipase and cellulases are widely used to produce laundry detergents. Apart from their extremozymes, haloalkaliphilic cells are also used to remove several toxic compounds from various environmental samples^22^ and therefore have high potential for environmental bioremediation applications.

In conclusion, Lake Van is the largest endorheic lake among the soda lakes and this feature provides the potential for a possible indicator of anthropogenic changes such as irrigation and seasonal changes. For this reason, to determine the microbiota of the lake, performing metabarcoding analyzes by sampling from different locations, depths, samples and also from different seasons is important in terms of determining evolutionary relationships and conducting ecological monitoring.

## Supplementary Information

Supplementary Information.

## Data Availability

16S bacterial metabarcoding data have been deposited in the NCBI Sequence Read Archive (SRA) under BioProject Number PRJNA731666 with BioSample accessions SAMN19292477-SAMN19292537.
