# Supplementary material for: Microbial community of soda Lake Van as obtained from direct and enriched water, sediment and fish samples
Source: Sci Rep. 2021 Sep 15;11:18364. doi: 10.1038/s41598-021-97980-3 (PMC8443733; doi:10.1038/s41598-021-97980-3)
Supplement: Supplementary file 1 — Supplementary Information. [file 41598_2021_97980_MOESM1_ESM.docx]

**SUPPLEMENTARY INFORMATION**

**Microbial community of soda Lake Van as obtained from direct and enriched water, sediment and fish samples**

**Table S1.** Shannon diversity index of each analyzed DNA samples using SILVA NGS v138.1

| **Sample**  **Code** | **Shannon**  **Diversity Index** |
| --- | --- |
| Direct DNA extraction from water samples | |
| DWWS1 | 3.55 |
| DWWS2 | 9.34 |
| DWWS5 | 7.31 |
| DWWS6 | 9.38 |
| DNA extraction from pre-enriched water samples in BHI | |
| WWS1 | 5.89 |
| WWS2 | 6.15 |
| WWS3 | 6.73 |
| WWS5 | 7.06 |
| WWS6 | 4.67 |
| WWS7 | 6.78 |
| WWS8 | 4.77 |
| WWS9 | 4.42 |
| Direct DNA extraction from sediment samples | |
| WS1K | 10.01 |
| WS2K | 9.01 |
| WS3K | 9.29 |
| WS5K | 13.75 |
| WS6K | 7.94 |
| WS8K | 12.80 |
| WS9K | 3.92 |
| DNA extraction from pre-enriched sediment samples in buffered peptone water | |
| WS1 | 3.83 |
| WS2 | 8.21 |
| WS3 | 7.81 |
| WS5 | 5.39 |
| WS6 | 6.65 |
| WS8 | 5.63 |
| WS9 | 6.62 |
| DNA extraction from pre-enriched sediment samples  in buffered peptone water supplemented with lake water | |
| WS1W | 7.47 |
| WS2W | 6.42 |
| WS3W | 6.76 |
| WS5W | 8.77 |
| WS6W | 7.06 |
| WS8W | 6.81 |
| WS9W | 8.24 |
| WSWcontrol | 5.29 |
| DNA extraction from pre-enriched fish samples in BHI | |
| VB1 | 5.05 |
| VB2 | 4.86 |
| VB3 | 5.36 |
| VB4 | 4.33 |
| VB5 | 4.33 |
| VB6 | 5.15 |
| VB7 | 6.35 |
| VB8 | 3.73 |
| VB9 | 4.44 |
| VB10 | 5.14 |
| VB11 | 5.34 |
| VB12 | 5.07 |
| VB13 | 6.64 |
| VB14 | 4.76 |
| VB15 | 3.58 |
| VB16 | 5.49 |
| VB17 | 5.28 |
| VB18 | 6.28 |
| VB19 | 5.30 |
| VB20 | 6.70 |
| VB21 | 6.12 |
| VB22 | 6.03 |
| VB23 | 3.20 |
| VB24 | 3.62 |
| VB25 | 4.86 |
| VB26 | 5.04 |
| VB27 | 5.03 |


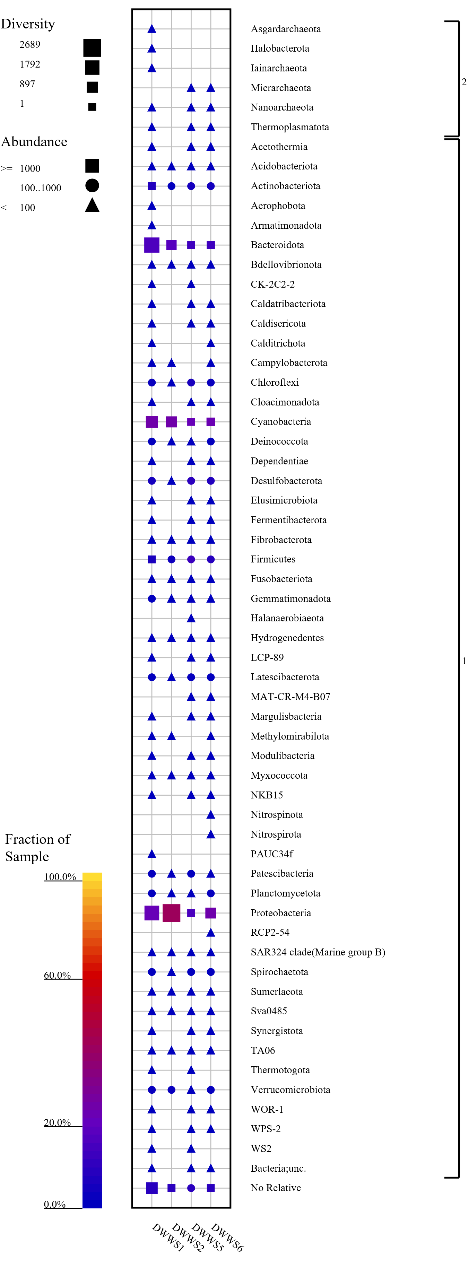


**Figure S1.** Taxonomic fingerprint of directly studied water samples of Lake Van at phylum-level (The figure was created using SILVA NGS 1.4 v138.1).


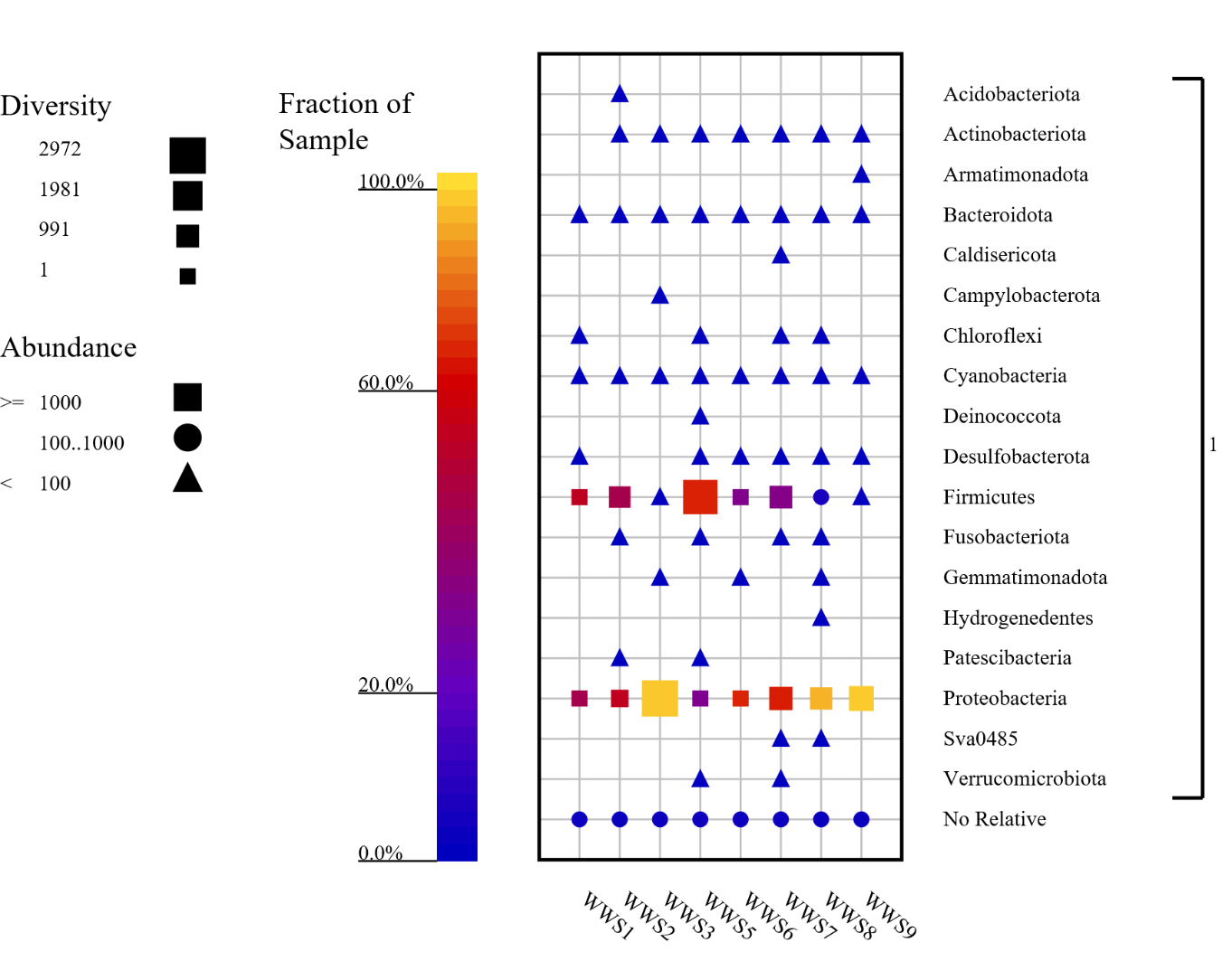


**Figure S2.** Taxonomic fingerprint of pre-enriched water samples of Lake Van at phylum-level (The figure was created using SILVA NGS 1.4 v138.1).

**
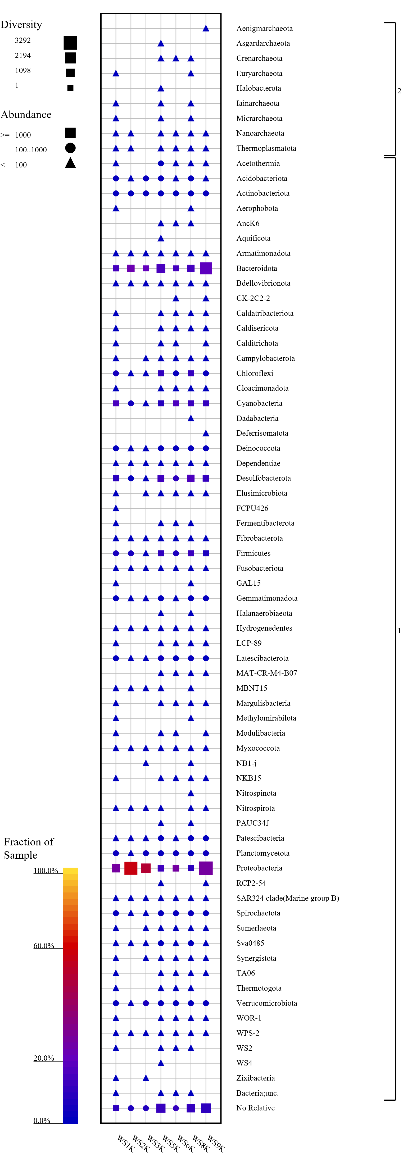
**

**Figure S3.** Taxonomic fingerprint of directly studied sediment samples of Lake Van at phylum-level (The figure was created using SILVA NGS 1.4 v138.1).

**
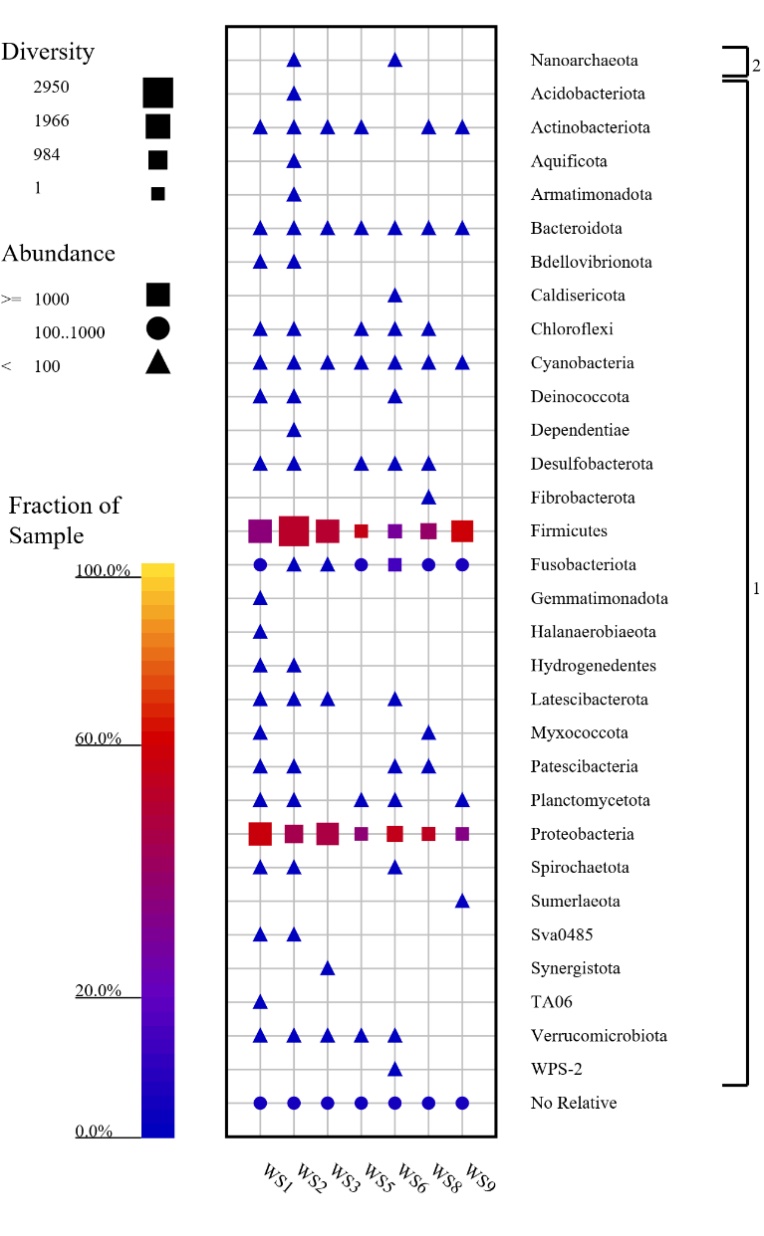
**

**Figure S4.** Taxonomic fingerprint of pre-enriched sediment samples in buffered peptone water of Lake Van at phylum-level (The figure was created using SILVA NGS 1.4 v138.1).

**
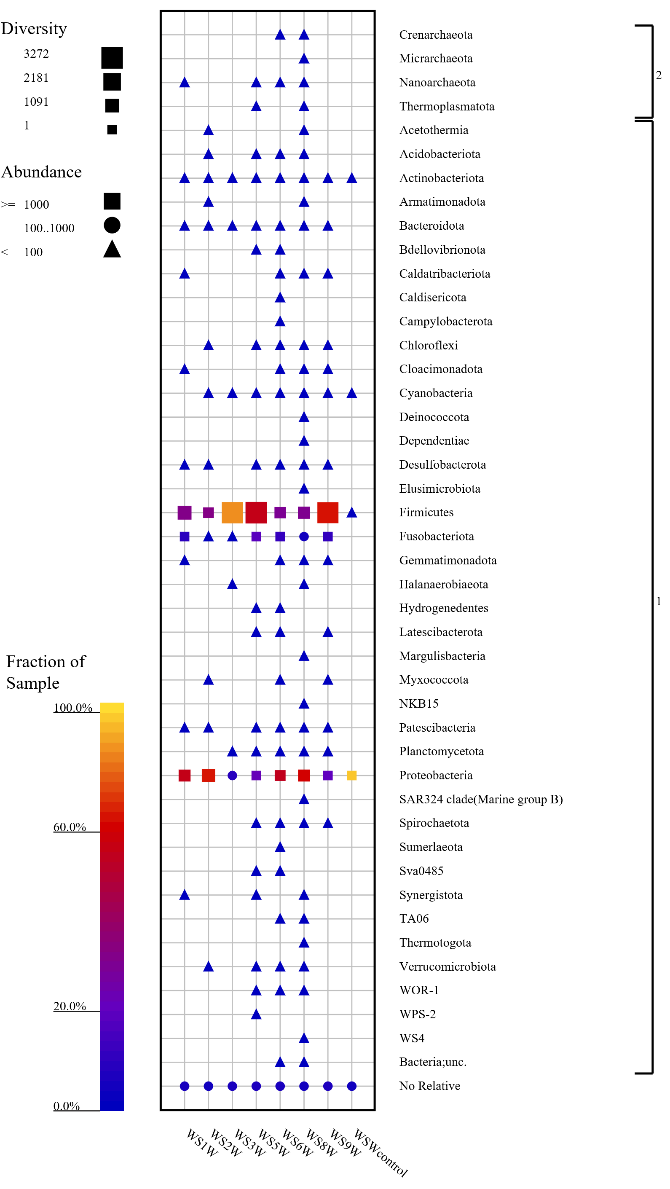
**

**Figure S5.** Taxonomic fingerprint of pre-enriched sediment samples in buffered peptone water supplemented with lake water of Lake Van at phylum-level (The figure was created using SILVA NGS 1.4 v138.1).

**
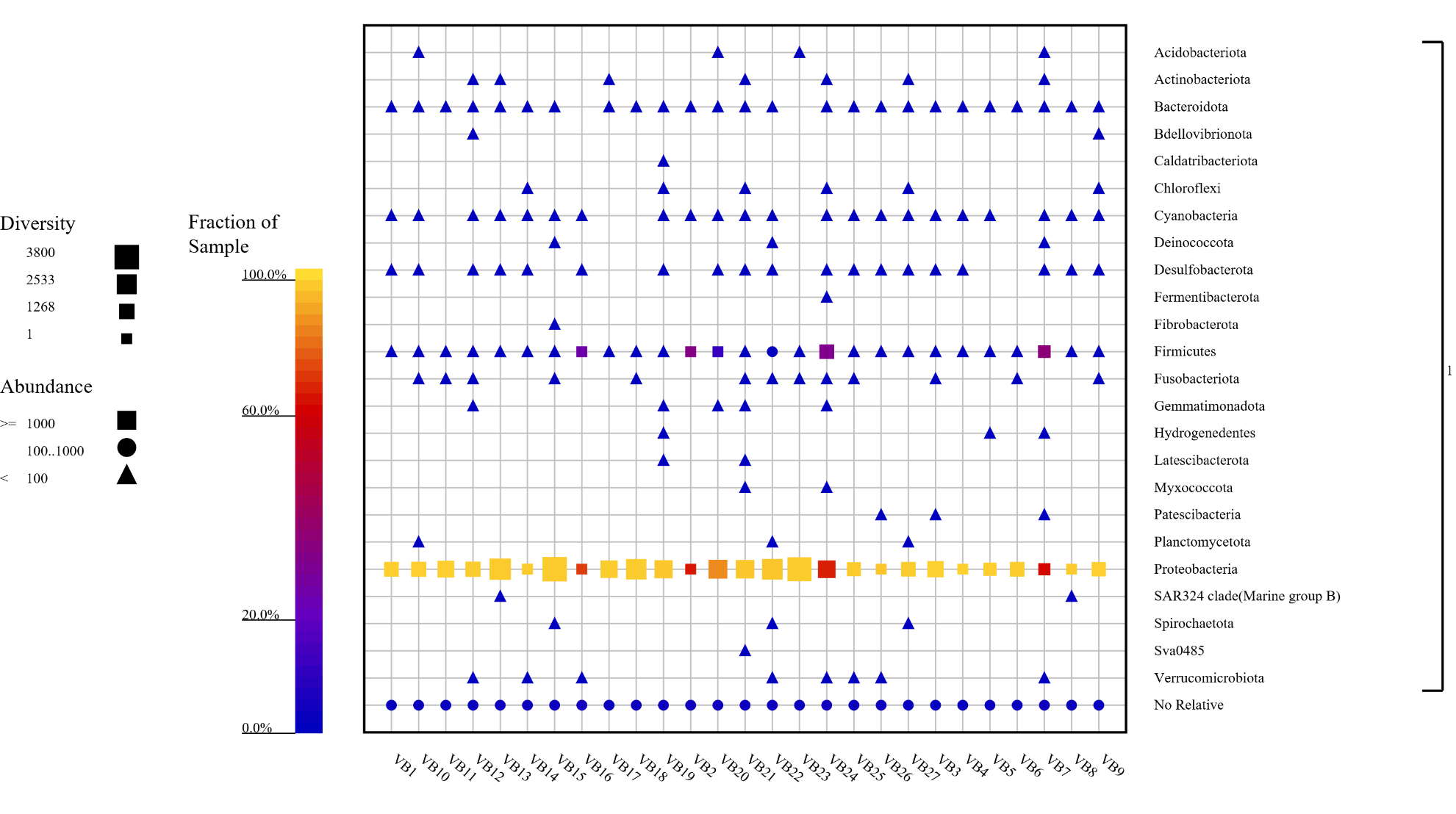
**

**Figure S6.** Taxonomic fingerprint of pre-enriched fish samples of Lake Van at phylum-level (The figure was created using SILVA NGS 1.4 v138.1).

**
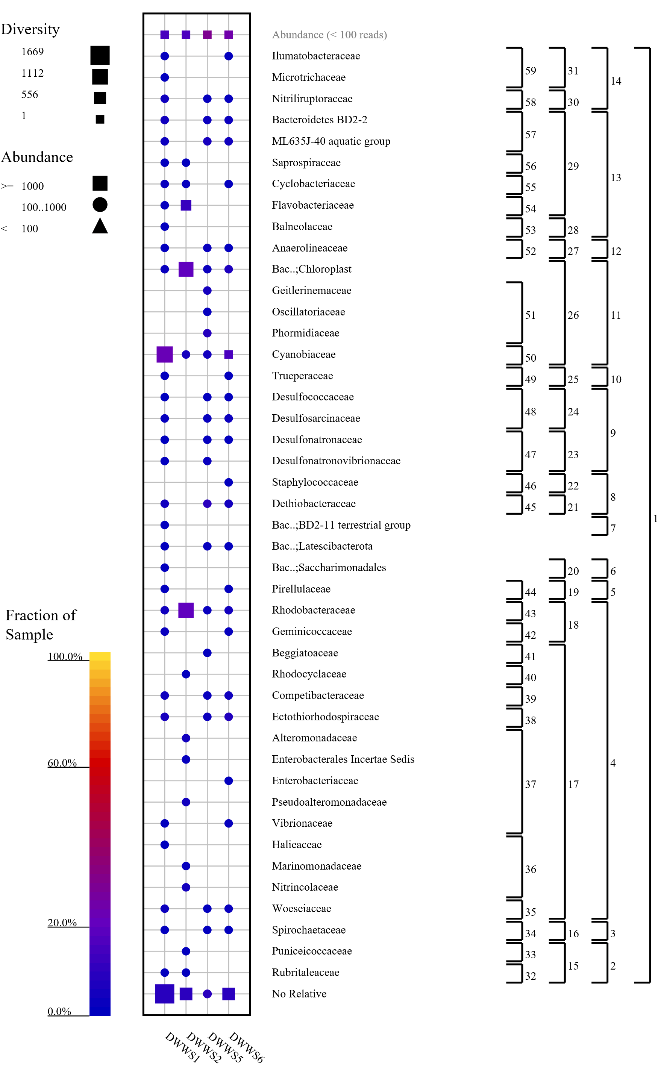
**

**Figure S7.** Taxonomic fingerprint of directly studied water samples of Lake Van at family-level (The figure was created using SILVA NGS 1.4 v138.1).

**
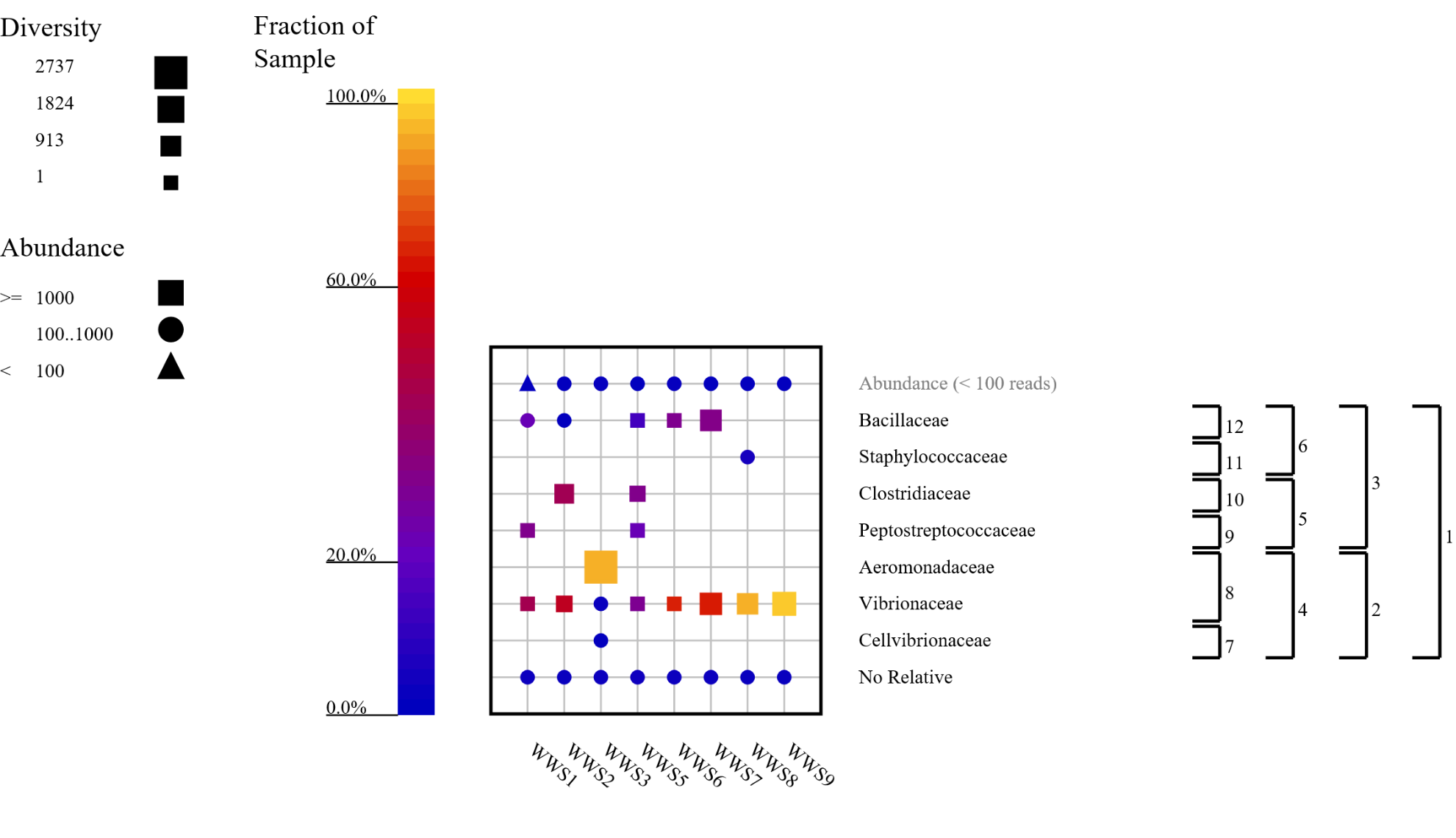
**

**Figure S8.** Taxonomic fingerprint of pre-enriched water samples of Lake Van at family-level (The figure was created using SILVA NGS 1.4 v138.1).

**
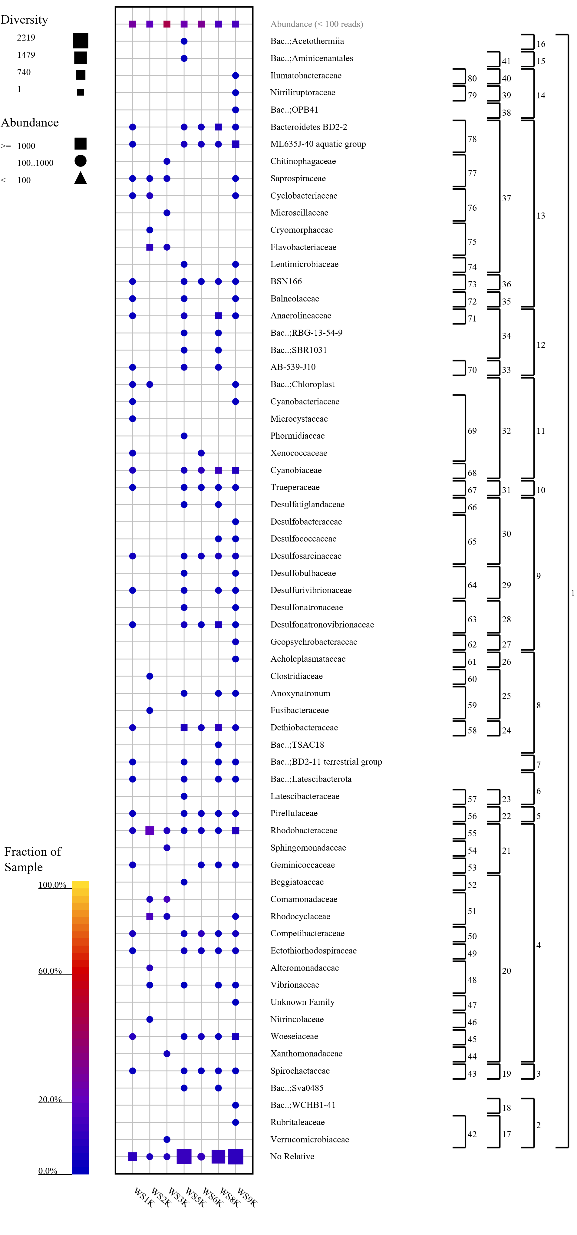
**

**Figure S9.** Taxonomic fingerprint of directly studied sediment samples of Lake Van at family-level (The figure was created using SILVA NGS 1.4 v138.1).

**
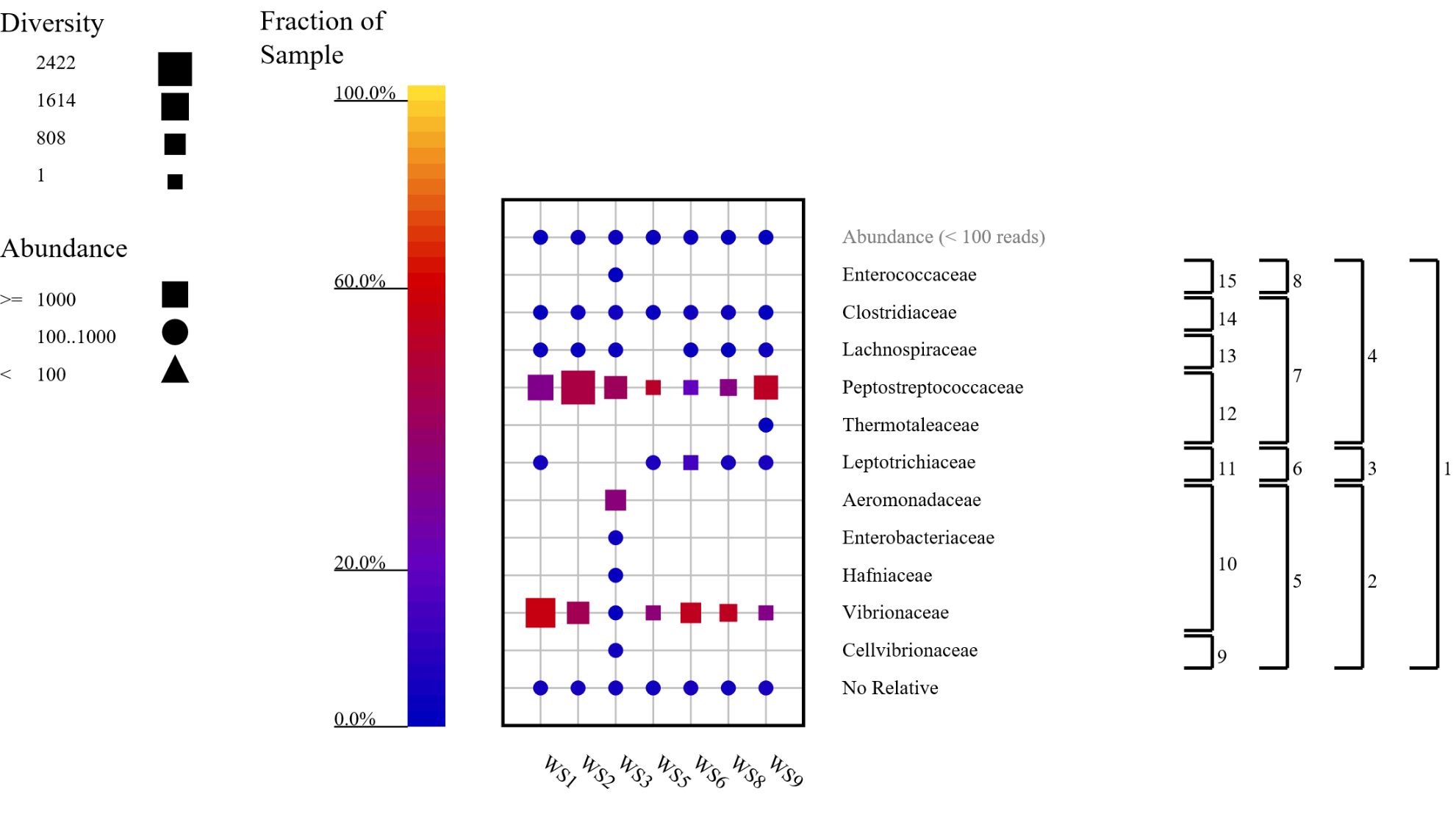
**

**Figure S10.** Taxonomic fingerprint of pre-enriched sediment samples in buffered peptone water of Lake Van at family-level (The figure was created using SILVA NGS 1.4 v138.1).

**
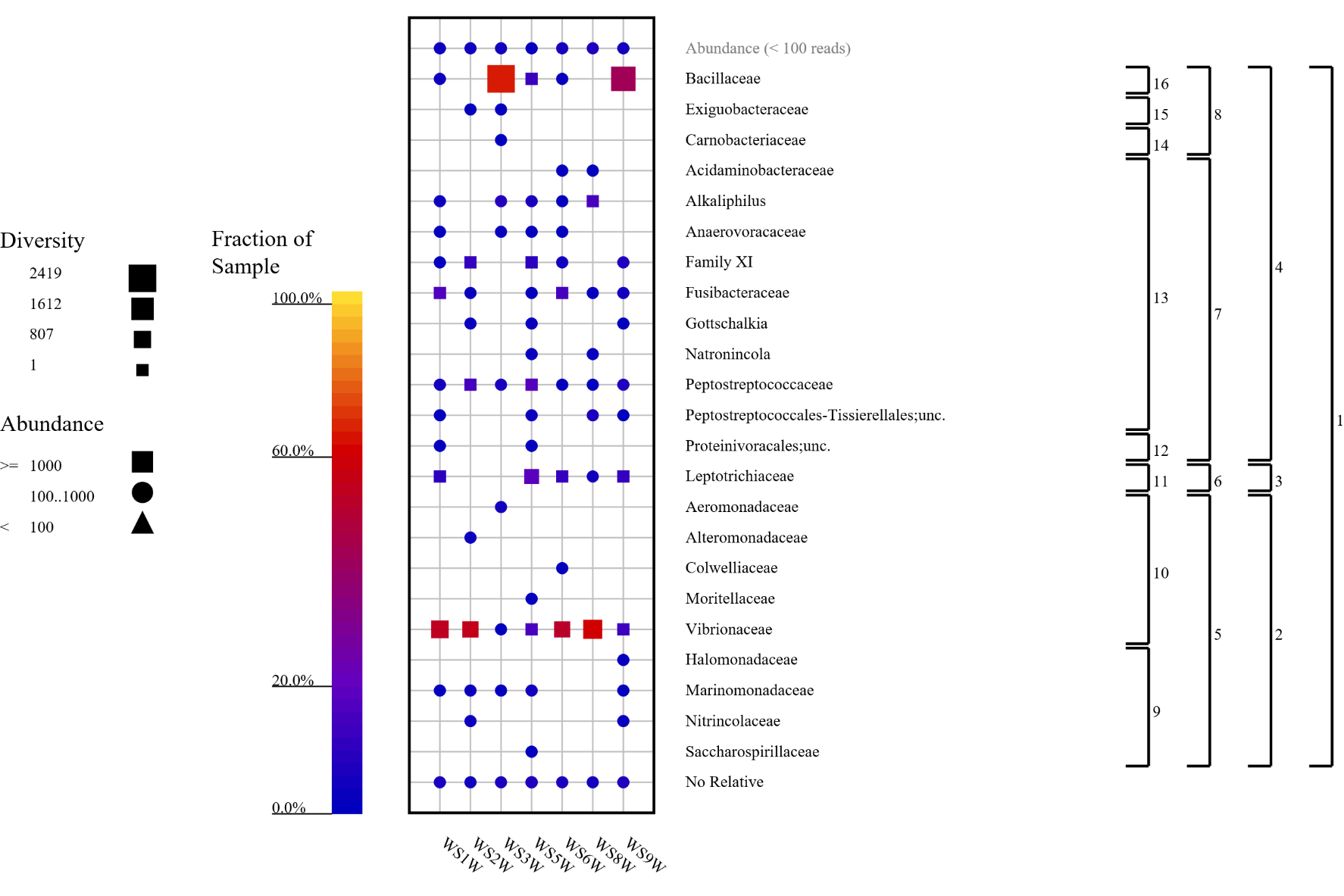
**

**Figure S11.** Taxonomic fingerprint of pre-enriched sediment samples in buffered peptone water supplemented with lake water of Lake Van at family-level (The figure was created using SILVA NGS 1.4 v138.1).

**
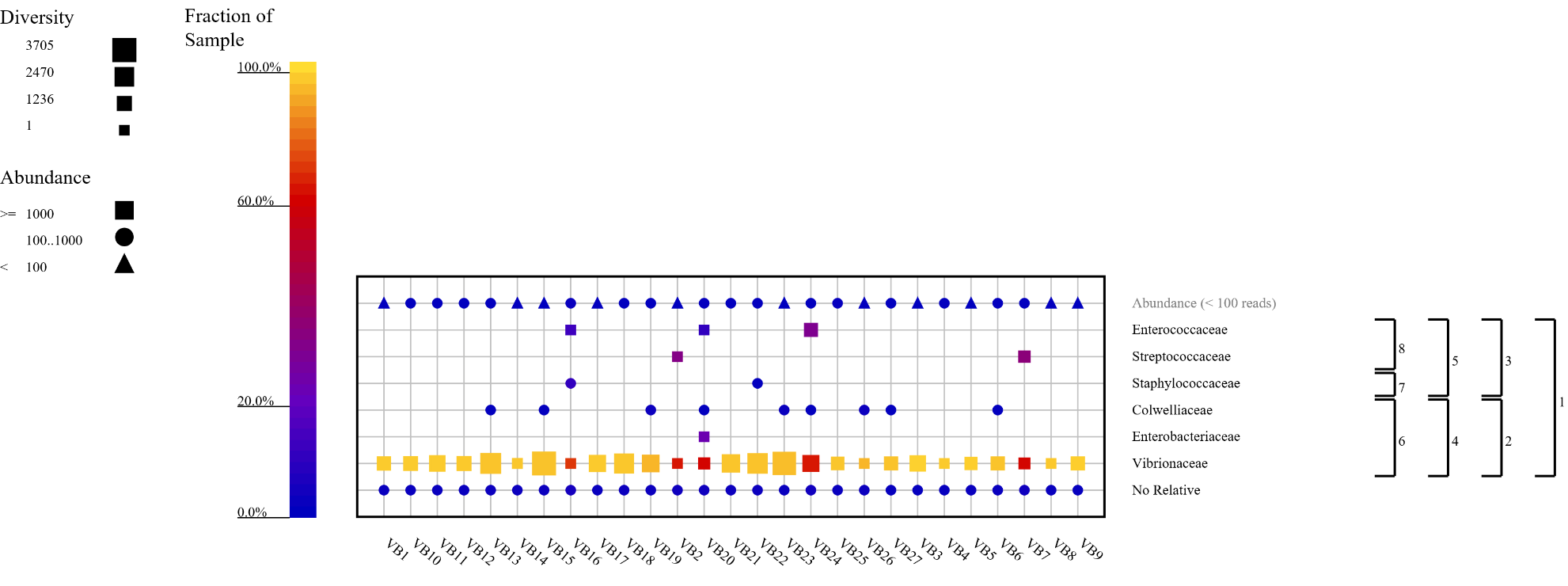
**

**Figure S12.** Taxonomic fingerprint of pre-enriched fish samples of Lake Van at family-level (The figure was created using SILVA NGS 1.4 v138.1).


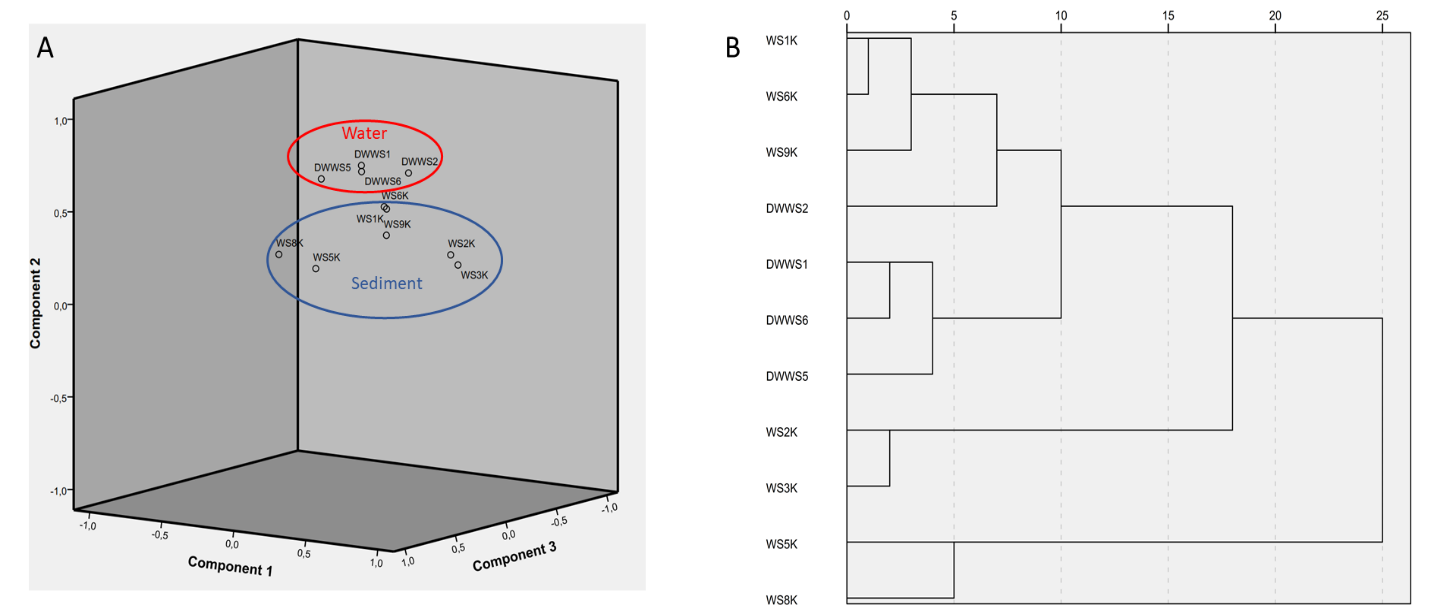


**Figure S13. A.** 3D view of PCA analysis of directly studied water (DWWS1-DWWS6) and sediment samples (WS1K-WS9K). **B.** Dendrogram showing average linkage within groups of samples (3D figure and dendrogram were generated using IBM SPSS Statistics program version 22.0).

**Figure S14.** PCA1 vs PCA2 of directly studied water (DWWS1-DWWS6, red) and sediment samples (WS1K-WS9K, blue) (Figure was generated using IBM SPSS Statistics program version 22.0).

**Figure S15.** PCA1 vs PCA3 of directly studied water (DWWS1-DWWS6, red) and sediment samples (WS1K-WS9K, blue) (Figure was generated using IBM SPSS Statistics program version 22.0).

**Figure S16.** PCA2 vs PCA3 of directly studied water (DWWS1-DWWS6, red) and sediment samples (WS1K-WS9K, blue) (Figure was generated using IBM SPSS Statistics program version 22.0).


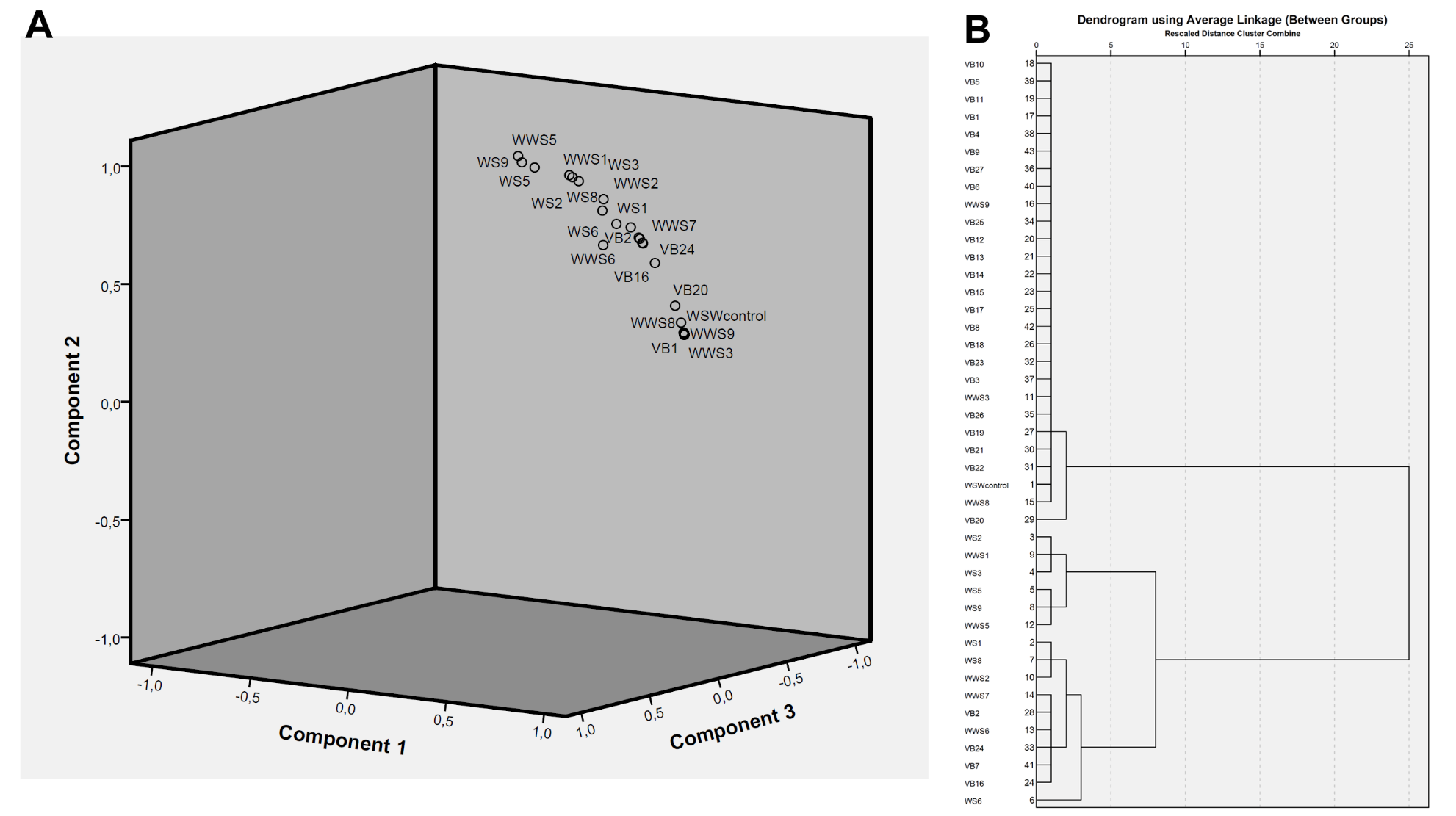


**Figure S17. A.** 3D view of PCA analysis of pre-enriched water (WWS1-WWS9), sediment (WS1-WS9, WS1W-WS9W), and fish samples (VB1-VB27). **B.** Dendrogram showing single linkage of samples (3D figure and dendrogram were generated by using IBM SPSS Statistics program version 22.0).


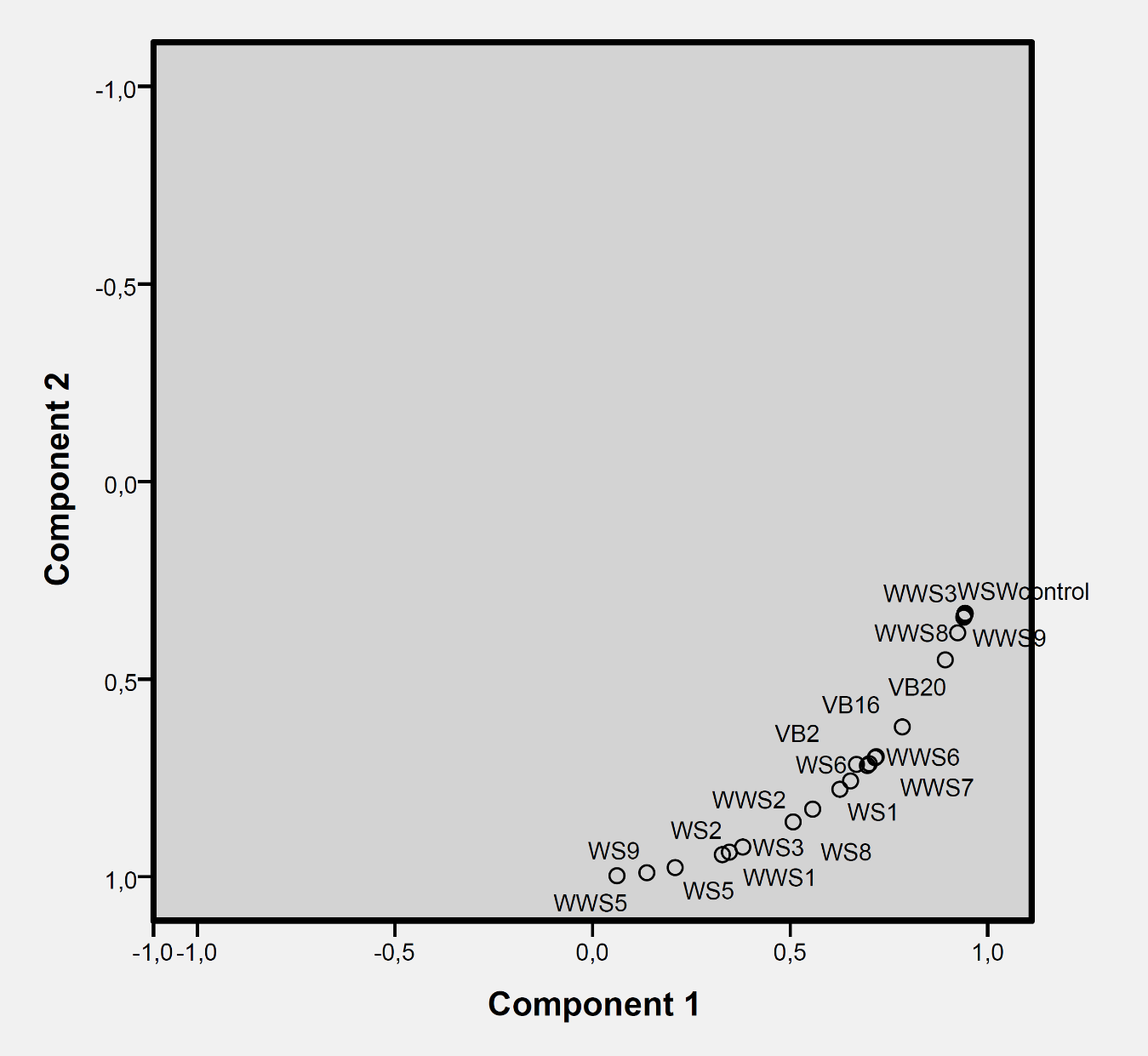


**Figure S18.** PCA1 vs PCA2 of pre-enriched water, sediment, and fish samples. (Figure was generated using IBM SPSS Statistics program version 22.0).

**
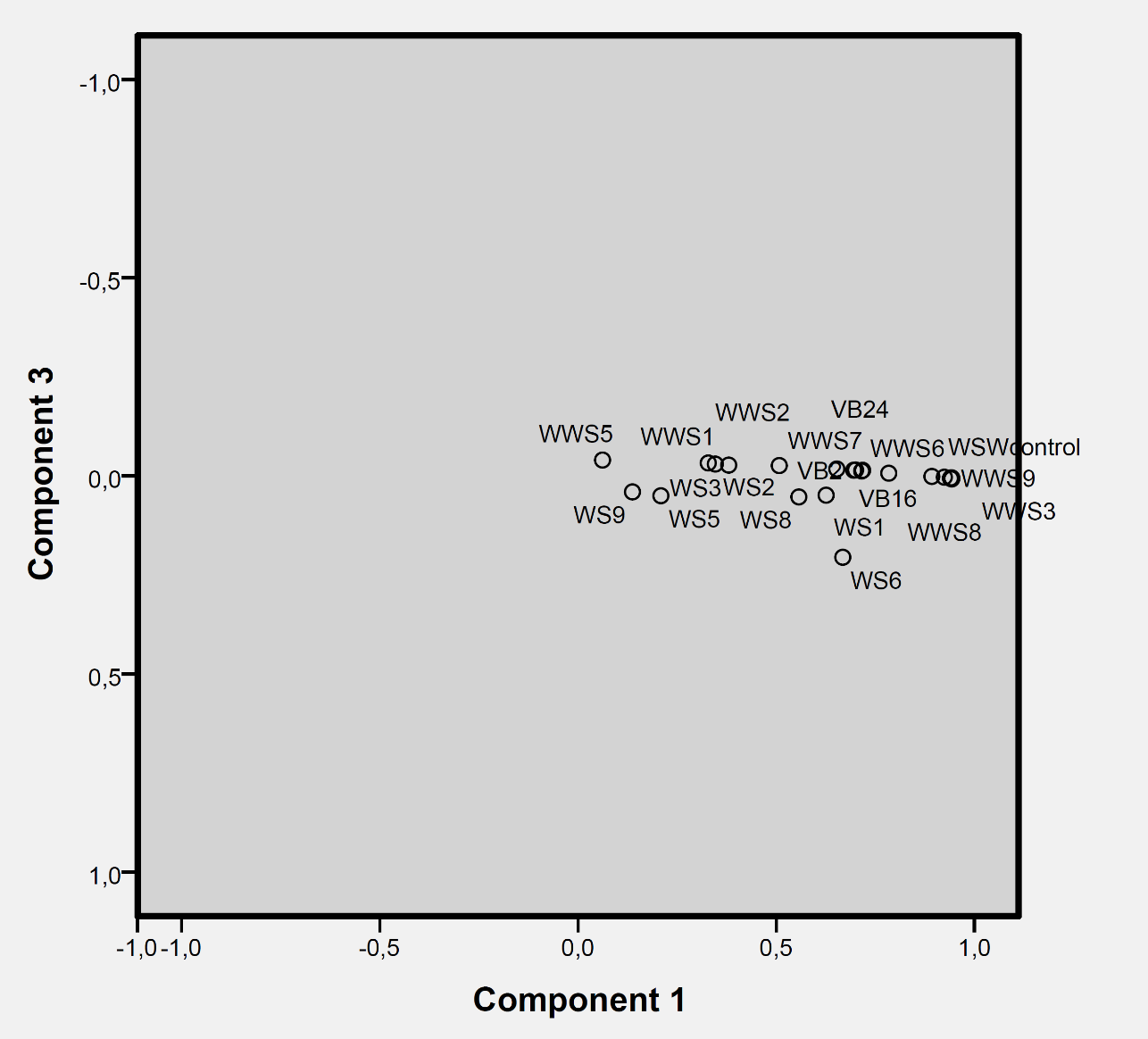
**

**Figure S19.** PCA1 vs PCA3 of pre-enriched water, sediment, and fish samples. (Figure was generated using IBM SPSS Statistics program version 22.0).

**
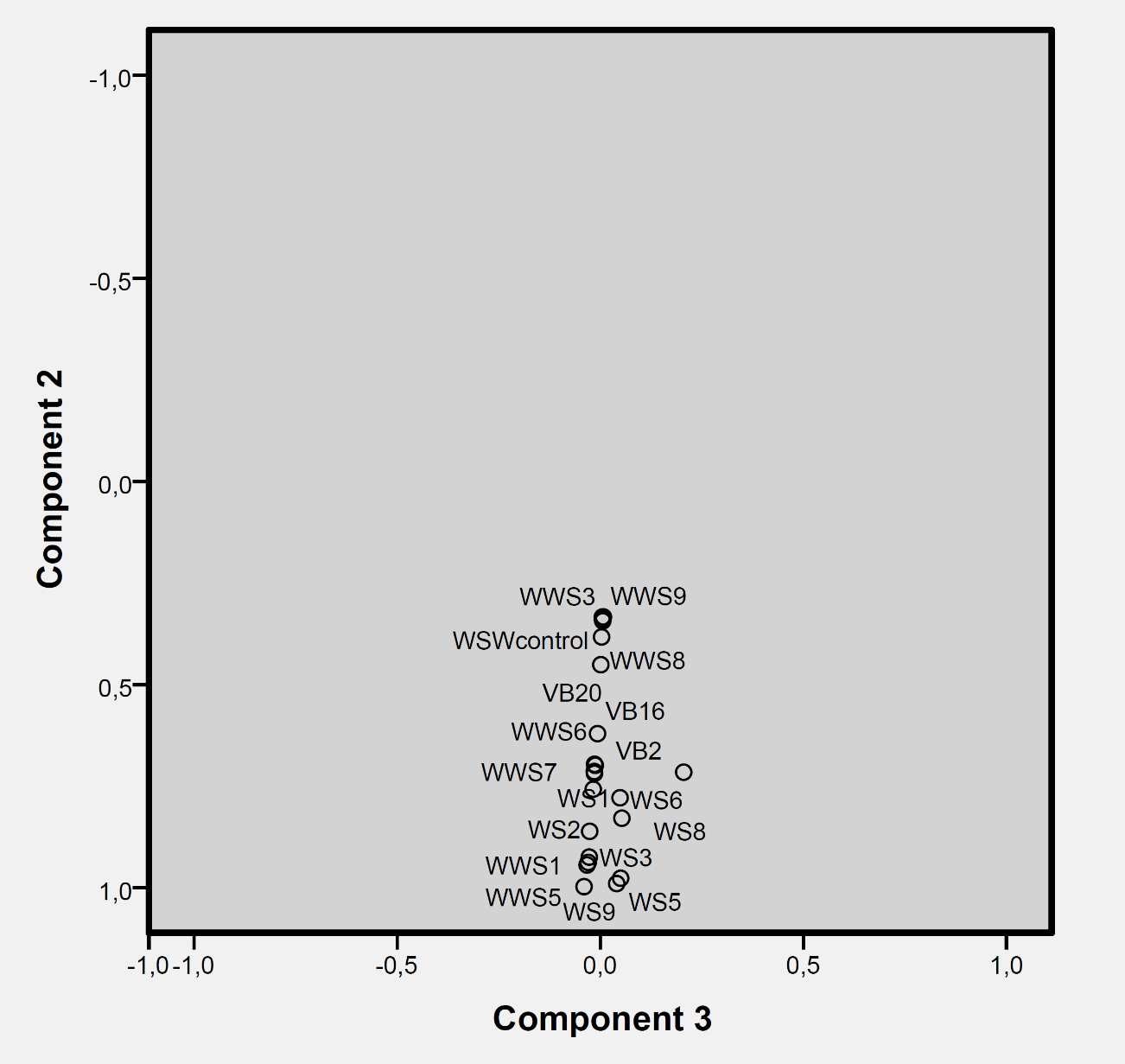
**

**Figure S20.** PCA2 vs PCA3 of pre-enriched water, sediment, and fish samples. (Figure was generated using IBM SPSS Statistics program version 22.0).
